# Intranasal vaccination with lipid-conjugated immunogens promotes antigen transmucosal uptake to drive mucosal and systemic immunity

**DOI:** 10.1126/scitranslmed.abn1413

**Published:** 2022-07-20

**Authors:** Brittany L. Hartwell, Mariane B. Melo, Peng Xiao, Ashley A. Lemnios, Na Li, Jason Y.H. Chang, Jingyou Yu, Makda S. Gebre, Aiquan Chang, Laura Maiorino, Crystal Carter, Tyson J. Moyer, Neil C. Dalvie, Sergio A. Rodriguez-Aponte, Kristen A. Rodrigues, Murillo Silva, Heikyung Suh, Josetta Adams, Jane Fontenot, J. Christopher Love, Dan H. Barouch, Francois Villinger, Ruth M. Ruprecht, Darrell J. Irvine

**Affiliations:** 1 Koch Institute for Integrative Cancer Research, Massachusetts Institute of Technology, Cambridge, MA 02139, USA.; 2 Ragon Institute of Massachusetts General Hospital, Massachusetts Institute of Technology and Harvard University, Cambridge, MA 02139, USA.; 3 Consortium for HIV/AIDS Vaccine Development (CHAVD), Scripps Research Institute, La Jolla, CA 92037, USA.; 4 New Iberia Research Center, University of Louisiana at Lafayette, New Iberia, LA 70560, USA.; 5 Center for Virology and Vaccine Research, Beth Israel Deaconess Medical Center, Harvard Medical School, Boston, MA 02115, USA.; 6 Harvard Medical School, Boston, MA 02115, USA.; 7 Department of Chemical Engineering, Massachusetts Institute of Technology, Cambridge, MA 02139, USA.; 8 Department of Biological Engineering, Massachusetts Institute of Technology, Cambridge, MA 02139, USA.; 9 Harvard-MIT Health Sciences and Technology, Institute for Medical Engineering and Science, Massachusetts Institute of Technology, Cambridge, MA 02139, USA.; 10 Department of Biology, University of Louisiana at Lafayette, New Iberia, LA 70560 USA.; 11 Department of Materials Science and Engineering, Massachusetts Institute of Technology, Cambridge, MA 02139 USA.; 12 Howard Hughes Medical Institute, Chevy Chase, MD 20815 USA.

## Abstract

To combat the HIV epidemic and emerging threats such as SARS-CoV-2, immunization strategies are needed that elicit protection at mucosal portals of pathogen entry. Immunization directly through airway surfaces is effective in driving mucosal immunity, but poor vaccine uptake across the mucus and epithelial lining is a limitation. The major blood protein albumin is constitutively transcytosed bidirectionally across the airway epithelium through interactions with neonatal Fc receptors (FcRn). Exploiting this biology, here, we demonstrate a strategy of “albumin hitchhiking” to promote mucosal immunity using an intranasal vaccine consisting of protein immunogens modified with an amphiphilic albumin-binding polymer-lipid tail, forming amph-proteins. Amph-proteins persisted in the nasal mucosa of mice and nonhuman primates and exhibited increased uptake into the tissue in an FcRn-dependent manner, leading to enhanced germinal center responses in nasal-associated lymphoid tissue. Intranasal immunization with amph-conjugated HIV Env gp120 or SARS-CoV-2 receptor binding domain (RBD) proteins elicited 100- to 1000-fold higher antigen-specific IgG and IgA titers in the serum, upper and lower respiratory mucosa, and distal genitourinary mucosae of mice compared to unmodified protein. Amph-RBD immunization induced high titers of SARS-CoV-2–neutralizing antibodies in serum, nasal washes, and bronchoalveolar lavage. Furthermore, intranasal amph-protein immunization in rhesus macaques elicited 10-fold higher antigen-specific IgG and IgA responses in the serum and nasal mucosa compared to unmodified protein, supporting the translational potential of this approach. These results suggest that using amph-protein vaccines to deliver antigen across mucosal epithelia is a promising strategy to promote mucosal immunity against HIV, SARS-CoV-2, and other infectious diseases.

## INTRODUCTION

To combat long-standing epidemics such as HIV and emerging threats such as severe acute respiratory syndrome coronavirus 2 (SARS-CoV-2), immunization strategies are needed that can elicit systemic antibody responses and humoral immunity at mucosal portals of entry in tandem (1–6). Many pathogens, including HIV, SARS-CoV-2, influenza, rotavirus, and cholera, infect the host through mucosal surfaces and thus are thought to require engagement of both systemic and mucosal branches of the immune system, using a combination of immunoglobulin G (IgG) and IgA antibodies, for effective management and protection (1, 6, 7). Secretory IgA (SIgA) is the main humoral defense at mucosal tissue sites (4) and plays a particularly important role in providing protection through mechanisms such as immune exclusion, inhibition of transcytosis, and direct neutralization of pathogens (8, 9). Establishment of antigen-specific SIgA antibodies at mucosal surfaces provides a frontline defense that can help prevent infection and transmission (10). With HIV, where 90% of transmissions occur through mucosal routes, induction of mucosal IgA responses, in combination with systemic IgG, has been found to be effective in promoting protection against mucosal simian-human immunodeficiency virus (SHIV) challenge in primates (11, 12). Similarly, SARS-CoV-2 clinical studies have shown that mucosal IgA exhibits potent neutralization and is a strong correlate of protection against the virus, which primarily infects cells in the upper and lower respiratory mucosa (13, 14).

Traditional parenteral immunization regimens typically elicit poor mucosal immunity. By contrast, vaccination at mucosal surfaces, which initiates immune responses in mucosa-associated lymphoid tissues (MALTs), is known to be a very effective strategy to promote protective immunity at barrier tissues; this is due to programming of mucosa-specific lymphocyte function and tissue homing at these sites (1, 3). Priming of mucosal T and B lymphocytes takes place in MALT inductive sites, such as the nasal-associated lymphoid tissue (NALT) and gut-associated lymphoid tissue (GALT) (3, 15, 16). Here, through a property of the “common mucosal immune system,” antigen priming can induce expression of homing markers that lead activated antigen-specific T cells, B cells, and plasma cells to migrate to other local or distal mucosal effector sites (2, 3, 7, 17). The location of antigen exposure determines which homing markers are expressed, dictating the homing destination and ultimate effector site. Typically, the strongest response is elicited at the site of antigen exposure and in the most anatomically adjacent mucosal tissue. For example, cells that experience antigen priming in the NALT acquire chemokine receptors and integrins (such as CCR10 and α_4_β_1_) that can home to both the respiratory tract and genitourinary tract; thus, intranasal immunization is able to establish humoral responses at both mucosal sites (2, 17).

Although well motivated by the biology of mucosal immunity, delivery of vaccine components across mucosal barriers has been a major challenge for mucosal vaccine development (1–3). Vaccine uptake into the underlying mucosal immune compartment is impeded by multiple factors, including potential rapid antigen loss due to degradation by proteolytic enzymes and acidic conditions at mucosal surfaces, high rates of mucociliary clearance, and the lack of diffusive uptake across the tight junctions of the epithelial monolayer (18–20). Only a small number of mucosal vaccines have reached licensure, all of which, except the inactivated oral cholera vaccine, are based on live attenuated pathogens that naturally infect mucosal surfaces; these include the oral polio vaccine or the intranasal influenza type A/B vaccine (FluMist) (3, 21, 22). However, live attenuated vaccines often face manufacturing challenges, poor stability, and safety concerns. These challenges have been addressed in parenteral vaccines by a focus on recombinant protein- or polysaccharide-based subunit vaccines that are safe, stable, and highly manufacturable, but subunit vaccines have historically exhibited poor immunogenicity and short-lived responses when applied to mucosal barriers, due, in large part, to challenges of delivery and poor uptake (3). Development of technologies to overcome barriers to mucosal delivery while meeting safety and efficacy requirements of prophylactic vaccines remains an urgent unmet need.

We previously developed a vaccine platform that uses endogenous albumin as a chaperone to enhance lymph node trafficking of peptide antigens or molecular adjuvants after parenteral injection. One of albumin’s primary functions in vivo is to serve as a fatty acid transporter because albumin bears seven different lipid-binding pockets (23, 24). By conjugating peptides or Toll-like receptor agonist adjuvants to an amphiphilic albumin-binding lipid tail (forming an “amph-vaccine”), important changes to the pharmacokinetic behavior of these vaccine components can be achieved. First, after injection, the lipid tail of amph-vaccines associates with endogenous albumin present in the interstitial fluid at the injection site, causing the conjugates to be efficiently redirected to lymphatic vessels and draining lymph nodes following the convection path of albumin (whereas unmodified peptides disperse into the blood where they are rapidly diluted and degraded) (25). Second, upon reaching the dense cellular microenvironment of lymph nodes, the lipid tails of amph-peptides insert into cell membranes, promoting prolonged antigen retention in the draining lymphoid tissue (26, 27). By optimizing the structure of the lipid tail and length of poly(ethylene glycol) (PEG) spacer in these molecules, the pharmacokinetics of amph-peptides were substantially altered compared to soluble peptide vaccines, leading to as much as 30-fold enhancements in systemic T cell responses and antitumor immunity after parenteral immunization (25, 28, 29).

In addition to constitutive trafficking from blood to tissues to lymph, albumin is also bidirectionally transported across mucosal barriers by interactions with the neonatal Fc receptor (FcRn) expressed by mucosal epithelial cells. The FcRn has received attention as a “mucosal gateway” for improving drug uptake across the mucosal epithelium in nasopharyngeal, pulmonary, and gastrointestinal tissues (7, 30–32). It is widely expressed on mucosal epithelial cells in adult animals and humans, where it plays an essential role in recycling IgG and albumin through bidirectional transcytosis of both molecules (33–35). We hypothesized that albumin-binding amph-vaccines might be capable of FcRn-mediated uptake across the nasal mucosa, enabling higher concentrations of antigen to reach the NALT. In addition, we envisioned that membrane tethering of amph-immunogens could prolong the availability of antigen in the nasal passages and NALT tissue to promote local immune priming while avoiding systemic dissemination of antigen away from the site of action of locally coadministered mucosal adjuvants. Together, these two effects would be expected to promote stronger mucosal and systemic immunity.

Our prior studies of amph-vaccines focused on relatively low molar mass peptide antigens targeting T cell immunity. Given that most licensed vaccines are thought to operate through induction of protective antibody responses (36, 37), here, we tested these ideas using 10-fold larger amph-protein immunogens designed to elicit humoral immune responses against HIV or SARS-CoV-2. We found that amph-protein immunogens show enhanced persistence and uptake across the nasal mucosa compared to unmodified antigens, leading to increased germinal center (GC) and follicular helper T cell (Tfh) responses in the NALT. In mice, intranasal amph-protein immunization led to high titers of IgG and IgA in serum, upper and lower respiratory mucosa, and distal genitourinary mucosal sites, including the induction of substantial neutralizing antibody (nAb) responses to a SARS-CoV-2 receptor binding domain (RBD) immunogen. We further show that amph-protein immunization enhances vaccine uptake in the nasal passages of nonhuman primates and enhances IgG and IgA responses relative to soluble protein immunization. Together, these data suggest that this simple approach to altering the pharmacokinetics of vaccine immunogens is promising as a strategy to enhance both mucosal and systemic immunity elicited by intranasal immunization.

## RESULTS

### Protein antigen–amphiphile conjugates exhibit albumin binding and membrane insertion properties

**Fig. 1.**
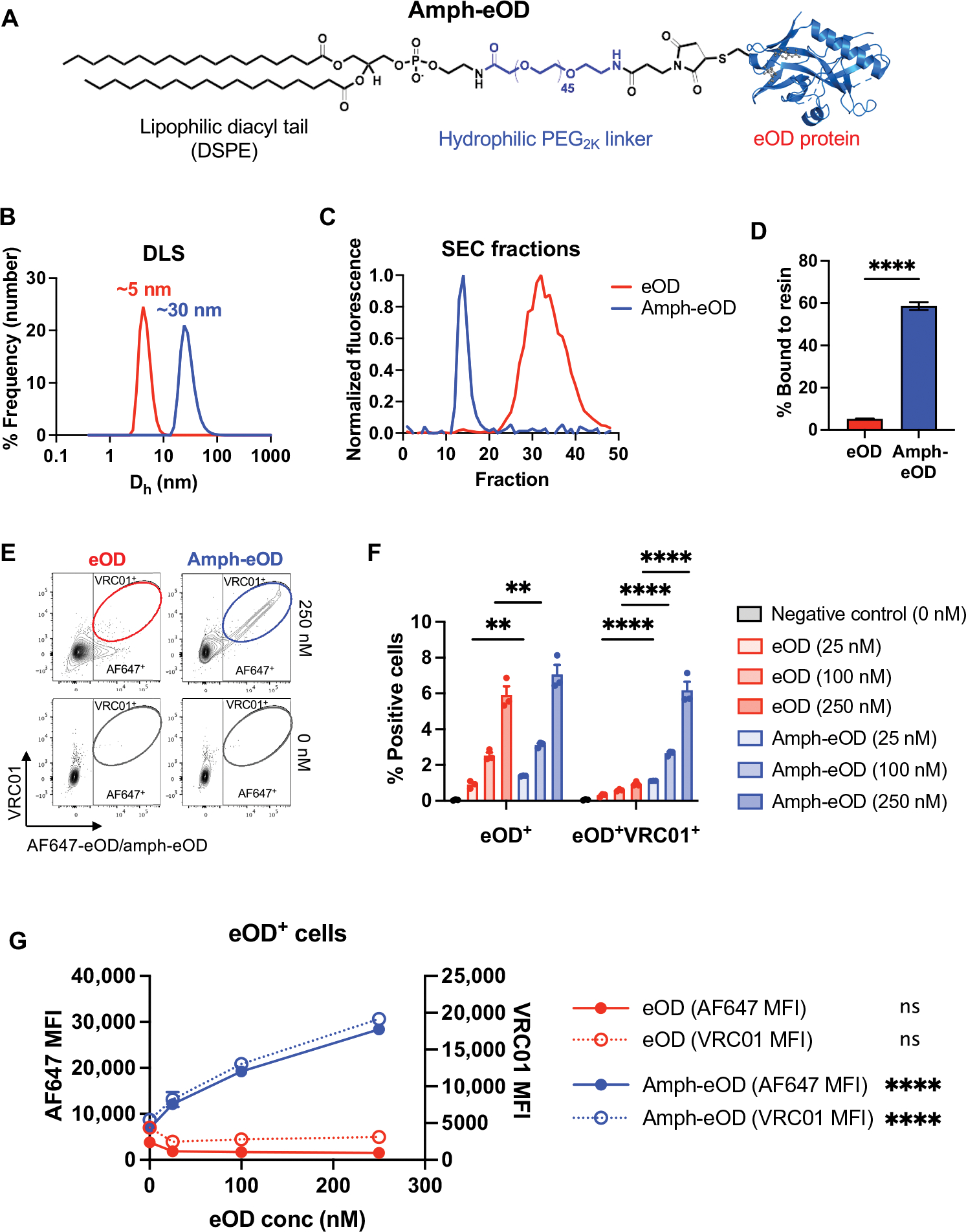
Synthesis of albumin-binding amphiphile-protein immunogen conjugates. (**A**) A schematic of amph-eOD structure is shown. (**B**) Dynamic light scattering (DLS) analysis of eOD and amph-eOD is shown as number-weighted % frequency. *D*_h_, hydrodynamic diameter. (**C**) Size exclusion chromatography (SEC) profiles of eOD and amph-eOD are shown. (**D**) AF647-eOD or AF647-amph-eOD protein were incubated with albumin-functionalized agarose resin at 37°C, and the quantity of each protein bound to the resin after 2 hours was quantified. Statistical significance was determined by unpaired *t* test. (**E** to **G**) Fluorescent eOD or amph-eOD were incubated with murine C57BL/6 splenocytes for 1 hour at 37°C at a range of concentrations and then washed and stained with fluorescent VRC01 antibody. (E) Representative flow cytometry plots are shown of eOD/amph-eOD and VRC01 binding to the cells. (F) The percentage of cells positive for eOD alone or double positive for eOD and VRC01 was quantified; statistical significance was determined by two-way ANOVA followed by Sidak’s post hoc test. (G) Mean fluorescence intensity (MFI) of eOD and VRC01 is shown as a function of eOD concentration; statistically significant nonzero slope was determined by simple linear regression. All data are presented as means ± SEM. **P < 0.01 and ****P < 0.0001; ns, not significant.

To assess whether appending an albumin-binding moiety to subunit protein vaccine antigens could alter antigen uptake across the nasal mucosa, we first synthesized conjugates of an HIV envelope (Env) protein immunogen linked to a PEG–1,2-distearoyl-sn-glycero-3-phosphoethanolamine (DSPE) amphiphile. We previously demonstrated that this PEG-lipid binds to albumin with an equilibrium dissociation constant (*K*_D_) of about 125 nM (25). As a test bed antigen for this concept, we selected the Env immunogen eOD-GT8 (gp120 engineered outer domain–germ line–targeting immunogen 8, hereafter eOD), a roughly 25-kDa germ line–targeting antigen that was recently shown to successfully prime VRC01-class HIV broadly neutralizing antibody responses in a phase 1 clinical trial (38–41). eOD was fused at the C terminus with the pan human leukocyte antigen DR–binding epitope (PADRE) peptide and a terminal free cysteine was introduced at the N terminus to enable coupling to maleimide-functionalized PEG_2K_-DSPE through a thioether linkage (fig. S1, A and B). The resulting amph-eOD (Fig. 1A) formed about 30-nm-diameter micelles in aqueous solution (Fig. 1B), facilitating purification from unreacted eOD by size exclusion chromatography (Fig. 1C).

We previously showed that PEG-DSPE coupling to small peptide antigens endows the conjugates with the ability to bind to albumin and to also interact with cell membranes, altering in vivo trafficking behavior (25, 26). To evaluate whether the amphiphile tail could similarly alter the behavior of much larger protein immunogens, we first incubated fluorescently labeled amph-eOD with an albumin-functionalized agarose resin for 2 hours at 37°C followed by separation of the resin and measurement of protein remaining in solution. Sixty percent of added amph-eOD bound to the albumin-resin versus less than 5% of unmodified eOD (Fig. 1D). Next, we assessed the interaction of amph-eOD with lymphocytes. Titrated concentrations of Alexa Fluor 647 (AF647) dye–labeled eOD or amph-eOD were added to mouse splenocytes in 10% serum at 37°C and then stained extracellularly at 4°C with fluorescently labeled VRC01 monoclonal antibody to detect eOD coating the cell surfaces. Flow cytometry analysis revealed that both eOD and amph-eOD showed association with splenocytes within 1 hour, but amph-eOD showed more than 15-fold greater magnitude of uptake (Fig. 1, E to G, and fig. S2, A and B). Furthermore, the vast majority of cell-associated amph-eOD was localized on the cell surfaces, as revealed by VRC01 staining (Fig. 1, E to G). The percentage of eOD^+^VRC01^+^ double-positive cells increased proportionally with amph-eOD but not eOD concentration (Fig. 1, F and G). Thus, amph-protein conjugates exhibit albumin-binding and membrane insertion properties similar to previously studied amph-peptide conjugates, which we hypothesized would alter antigen trafficking and persistence in vivo.

### Amphiphile modification enhances uptake and retention of eOD antigen in the nasal cavity after intranasal immunization in mice

**Fig. 2.**
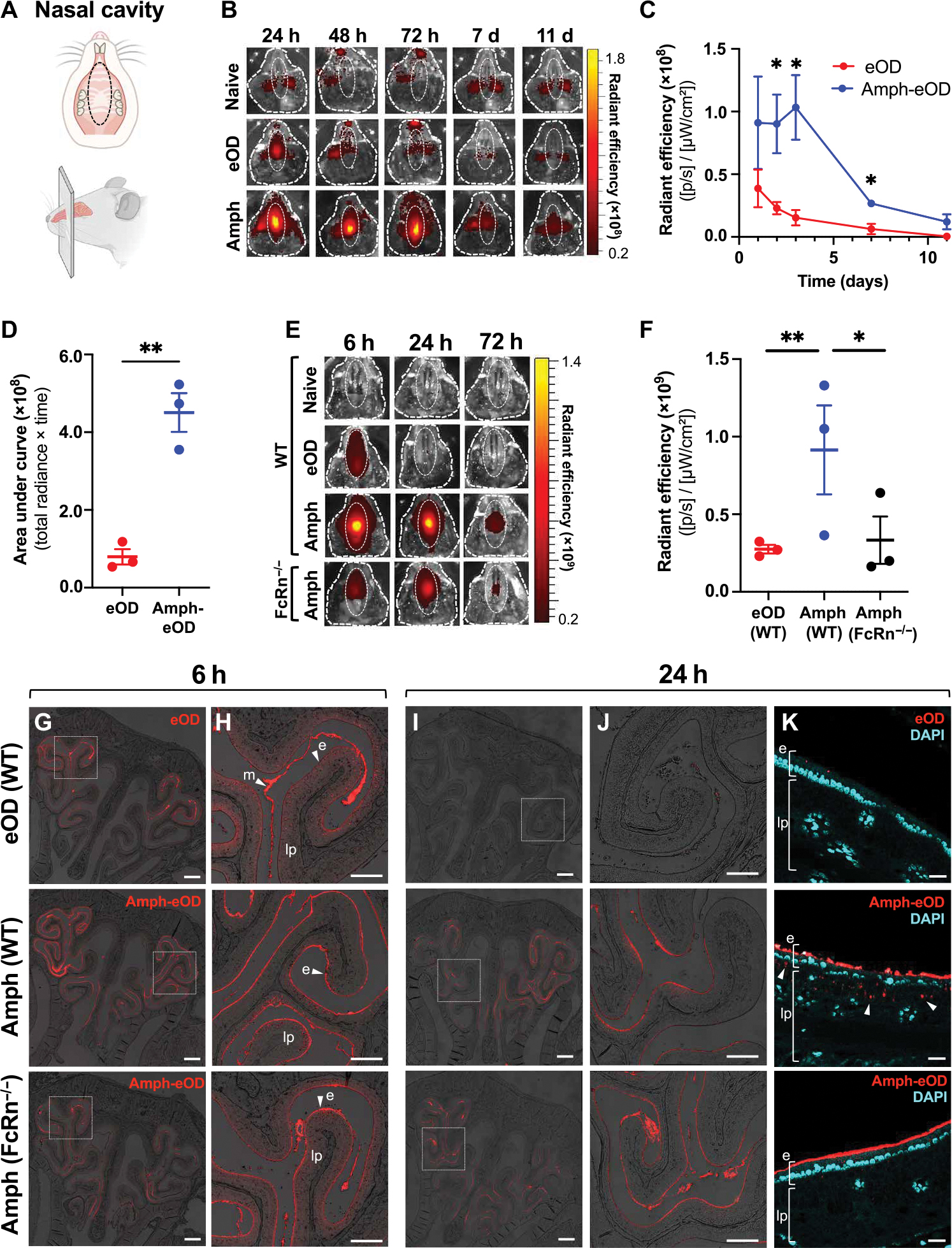
Amph-protein conjugates exhibit enhanced persistence in the nasal mucosa and transport across the mucosal surface. (**A**) Schematics illustrating (top) the ventral view of mouse upper palate and underside of top jaw, showing regions of interest (ROIs) used to quantify IVIS signals in (B) and (E), and (bottom) the sagittal view of mouse skull and nasal cavity showing approximate location of corresponding coronal cross sections used for histology in (G) and (H). (**B**) Representative IVIS images are shown of fluorescent signal in the nasal cavity of BALB/c mice (*n* = 3 animals per group) over time after intranasal administration of 5 μg of AF647-eOD or AF647-amph-eOD mixed with 5 μg of SMNP adjuvant. ROIs used to quantify IVIS signal are marked with dotted white oval. h, hour; d, day. (**C**) Quantified IVIS signals from (B) in nasal cavity over time are shown as average radiant efficiency. p, photon. Statistical significance was determined by unpaired *t* test at each time point. Data shown are from one representative of two independent experiments. (**D**) Quantified IVIS signal area under the curve (AUC; total radiance × time) from (C) was calculated. Statistical significance was determined by an unpaired *t* test. (**E**) Representative IVIS images show vaccine uptake and retention in the nasal cavity over time after intranasal administration of 5 μg of AF647-eOD or AF647-amph-eOD mixed with 5 μg of SMNP adjuvant in WT C57BL/6 versus FcRn^−/−^ mice (*n* = 3 animals per group). (**F**) Quantified IVIS signal from (E) is shown for the nasal cavities of WT versus FcRn^−/−^ mice at 6 hours after vaccination. Statistical significance was determined by two-way ANOVA followed by Tukey’s post hoc test. (**G** and **H**) Representative histology images of vaccine in nasal cavity in WT versus FcRn^−/−^ mice at 6 hours are shown. Images in (H) are higher magnification views of dashed areas marked in (G). Scale bars, 1 mm (G) and 500 μm (H). (**I** to **K**) Representative histology images of vaccine in nasal cavity in WT versus FcRn^−/−^ mice at 24 hours and immunization. Images in (J) are higher magnification views of dashed areas noted in (I). (K) High-magnification views stained with DAPI to identify the epithelial cell barrier; white arrows denote vaccine uptake. e marks epithelium, lp marks lamina propria, and m marks mucus. Scale bars, 1 mm (I), 500 μm (J), and 100 μm (K). All data are presented as means ± SEM. *P < 0.05 and **P < 0.01.

Albumin is transported bidirectionally across respiratory mucosal surfaces through interactions with FcRn (31, 32, 42). We thus hypothesized that amph-protein immunogens might show enhanced uptake across the nasal mucosal epithelium by using albumin as a noncovalent chaperone. To test this idea, we first assessed whether DSPE-PEG binding to albumin would inhibit its interaction with FcRn using an enzyme-linked immunosorbent assay (ELISA) to measure albumin binding to plate-bound FcRn. Incubation of albumin with fluorescein isothiocyanate–labeled DSPE-PEG at concentrations up to 1 μM showed no inhibition of albumin-FcRn binding (fig. S2C). We next investigated trafficking of fluorescent amph-eOD vaccine in the nasal cavity of mice over time after intranasal administration. Total vaccine uptake in the nasal cavity was quantified by in vivo imaging system (IVIS) measurement of fluorescence signal in a defined region of interest of the mouse snout over time (Fig. 2A, top) and was further characterized by histological imaging of cross sections of the nasal cavity (Fig. 2A, bottom). First, BALB/c mice were immunized intranasally with AF647-labeled eOD or amph-eOD mixed with saponin adjuvant; upper jaws were removed from the mouse snout, and the signal on the ventral side of the nasal cavity was quantified by IVIS over 11 days (Fig. 2B). Amph-eOD showed significant (*P* < 0.05) accumulation and persistence in the nasal cavity over 72 hours, with vaccine still detectable at 7 and 11 days after immunization (Fig. 2, B and C). By contrast, free eOD exhibited some initial signal at 24 hours (less than 40% of amph-eOD), which quickly decreased to background. Vaccine exposure assessed as area under the curve for the nasal fluorescence signal over time was about 5.7-fold greater for amph-eOD than eOD (*P* < 0.01; Fig. 2D). Furthermore, amph-eOD did not disseminate to reach the systemic compartment or distal lymphatic tissues; negligible vaccine accumulation was observed by IVIS in the spleen, liver, intestines, cervical lymph nodes, or mesenteric lymph nodes at 24 hours (fig. S3, A and B). We hypothesized that enhanced amph-vaccine persistence in the nasal cavity could be mediated by a combination of (i) the lipid tail promoting association with the epithelial cell surfaces and (ii) amphiphile binding to albumin in the mucus layer promoting FcRn-mediated transcytosis into the underlying nasal submucosa. IVIS imaging revealed rapid clearance of amph-eOD administered with adjuvant intranasally in FcRn^−/−^ mice compared to wild-type (WT) animals; amph-eOD persistence in the FcRn-deficient animals was similar to unmodified eOD in WT mice (Fig. 2, E to F).

To determine whether enhanced antigen persistence correlated with actual uptake into the nasal tissue, we imaged histological sections from the mid-point of the nasal passages (Fig. 2A). Confocal imaging revealed immediate qualitative differences in vaccine accumulation and uptake in the nasal cavity at 6 hours (Fig. 2, G to H). eOD was only faintly observed on the epithelial cell surface (“e”) and appeared instead to be primarily trapped at the top of the mucus layers (“m”) lining the airways (Fig. 2H). Conversely, amph-eOD predominantly accumulated at the epithelial surface overlying the lamina propria (“lp”) in WT mice, concentrating in the nasal turbinates. We also examined the amph-protein distribution in FcRn^−/−^ mice, which showed a trend toward about 50% lower concentration of albumin in nasal washes than WT animals, but still had detectable albumin present (fig. S3C). Amph-eOD also exhibited clear accumulation at the epithelial surface of FcRn^−/−^ mice (Fig. 2, G to H), which we attribute to the amphiphile tail’s ability to insert into cell membranes. At 24 hours after administration, eOD was nearly undetectable in the nasal cavity, whereas amph-eOD was still accumulated at the epithelial surface in WT and FcRn^−/−^ animals (Fig. 2, I and J). However, higher magnification imaging with 4′,6-diamidino-2-phenylindole (DAPI) staining to delineate the epithelium and underlying submucosa revealed clear pockets of amph-eOD uptake into the lamina propria in WT mice; this submucosal accumulation was absent in FcRn^−/−^ mice (Fig. 2K). These data suggest that association of eOD with epithelial cells is promoted by the DSPE lipid tail, but transport across the epithelial barrier is dependent on FcRn.

### Intranasal amph-eOD induces superior GC and Tfh cell responses in NALT in an FcRn-dependent manner

**Fig. 3.**
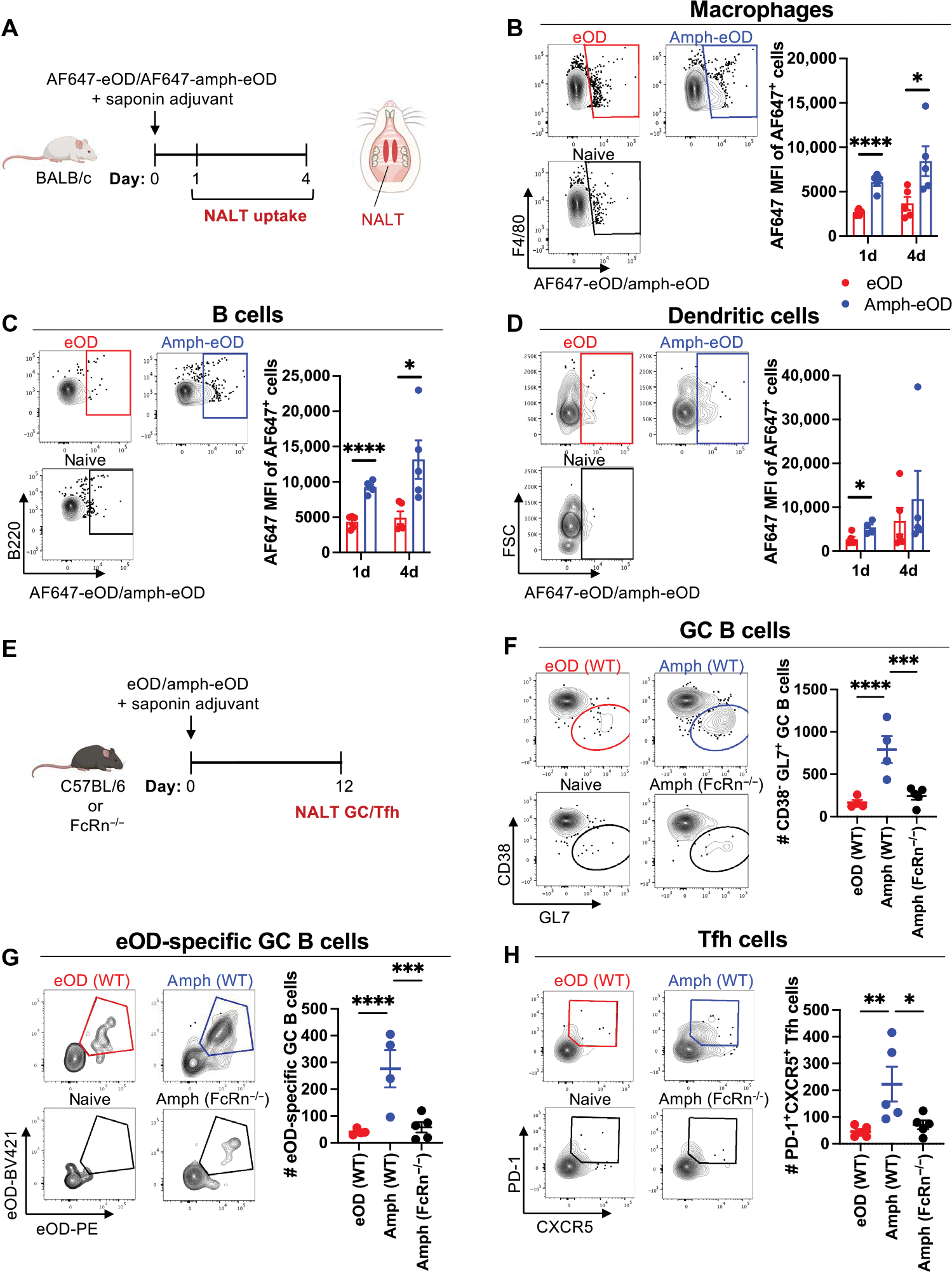
Amph-protein conjugates prime enhanced GC B cell and Tfh responses in the NALT in an FcRn-dependent manner. (**A** to **D**) Groups of BALB/c mice (*n* = 5 animals per group) were immunized intranasally with 10 μg of AF647-amph-eOD or AF647-eOD mixed with 5 μg of saponin adjuvant, and NALT tissue was isolated 1 or 4 days later for flow cytometry analysis of antigen uptake. (A) Schematics illustrating (left) experimental timeline and (right) NALT tissue location are shown. (B to D) Representative flow cytometry plots of eOD signal gating and mean fluorescence intensities are shown for F4/80^+^ macrophages (B), B cells (C), and CD11c^+^ dendritic cells (D). FSC, forward scatter. Statistical significance was determined by unpaired *t* tests. (**E** to **H**) Groups of C57BL/6 (WT) or FcRn^−/−^ mice (*n* = 5 animals per group) were immunized with 5 μg of eOD or amph-eOD mixed with 5 μg of saponin adjuvant; GC and Tfh responses were analyzed by flow cytometry on day 12. (E) The schematic shows experimental timeline. (F to H) Representative flow cytometry gating and enumeration of total GC B cells (F), antigen-specific GC B cells (G), and Tfh cells (H) are shown. Data shown are from one representative of two independent experiments. Statistical significance was determined by ordinary one-way ANOVA followed by Tukey’s post hoc test. All data are presented as means ± SEM. *P < 0.05, **P < 0.01, ***P < 0.001, and ****P < 0.0001.

We hypothesized that enhanced vaccine retention in the nasal cavity and increased uptake across the nasal mucosal epithelia would result in greater amounts of antigen reaching the NALT located on the dorsal side of the soft palate underlying the nasal passage, thereby priming a stronger local GC response. We thus investigated fluorescent eOD or amph-eOD accumulation and persistence in the NALT over time by flow cytometry after intranasal immunization (Fig. 3A). Amph-eOD accumulation in F4/80^+^ macrophages and B cells exceeded that of eOD both 1 and 4 days after immunization (Fig. 3, B and C, and fig. S4). Uptake in CD11c^+^MHCII^+^ dendritic cells was also greater for amph-eOD compared to eOD 1 day after immunization (Fig. 3D and fig. S4). These findings indicate that amph-eOD reaches the NALT and is taken up by key antigen-presenting cell (APC) populations to a greater extent than unmodified eOD.

To determine the impact of enhanced antigen delivery to the nasal lymphoid tissue on the initial stages of the adaptive immune response to eOD, we evaluated GC B cell and Tfh cell responses in the NALT 12 days after intranasal immunization with eOD and saponin adjuvant (Fig. 3E). Amph-eOD induced a greater GC response in the NALT of WT mice, both in terms of total GC B cells (4.8-fold) and eOD-binding antigen-specific GC B cells (6.8-fold) in comparison to soluble eOD immunization (Fig. 3, F and G, and fig. S5). These amplified responses were completely dependent on FcRn because amph-eOD immunization in FcRn^−/−^ animals elicited responses comparable to eOD in WT mice (Fig. 3, F and G, and fig. S5, B to E). These trends were mirrored in NALT Tfh responses; amph-eOD elicited greater Tfh responses compared to both eOD in WT mice and amph-eOD in FcRn^−/−^ mice (Fig. 3H and fig. S6, A to E) and induced greater overall activation of T cells [inducible costimulator (ICOS)^+^CD4^+^CD44^+^ T cells] compared to eOD in WT mice (*P* < 0.01) and amph-eOD in FcRn^−/−^ mice (*P* < 0.05; fig. S6, B and C). Thus, amph-conjugate immunization amplifies mucosal GC and T cell responses in a manner dependent on FcRn.

### Intranasal amph-eOD elicits robust systemic and mucosal antibody responses in mice

**Fig. 4.**
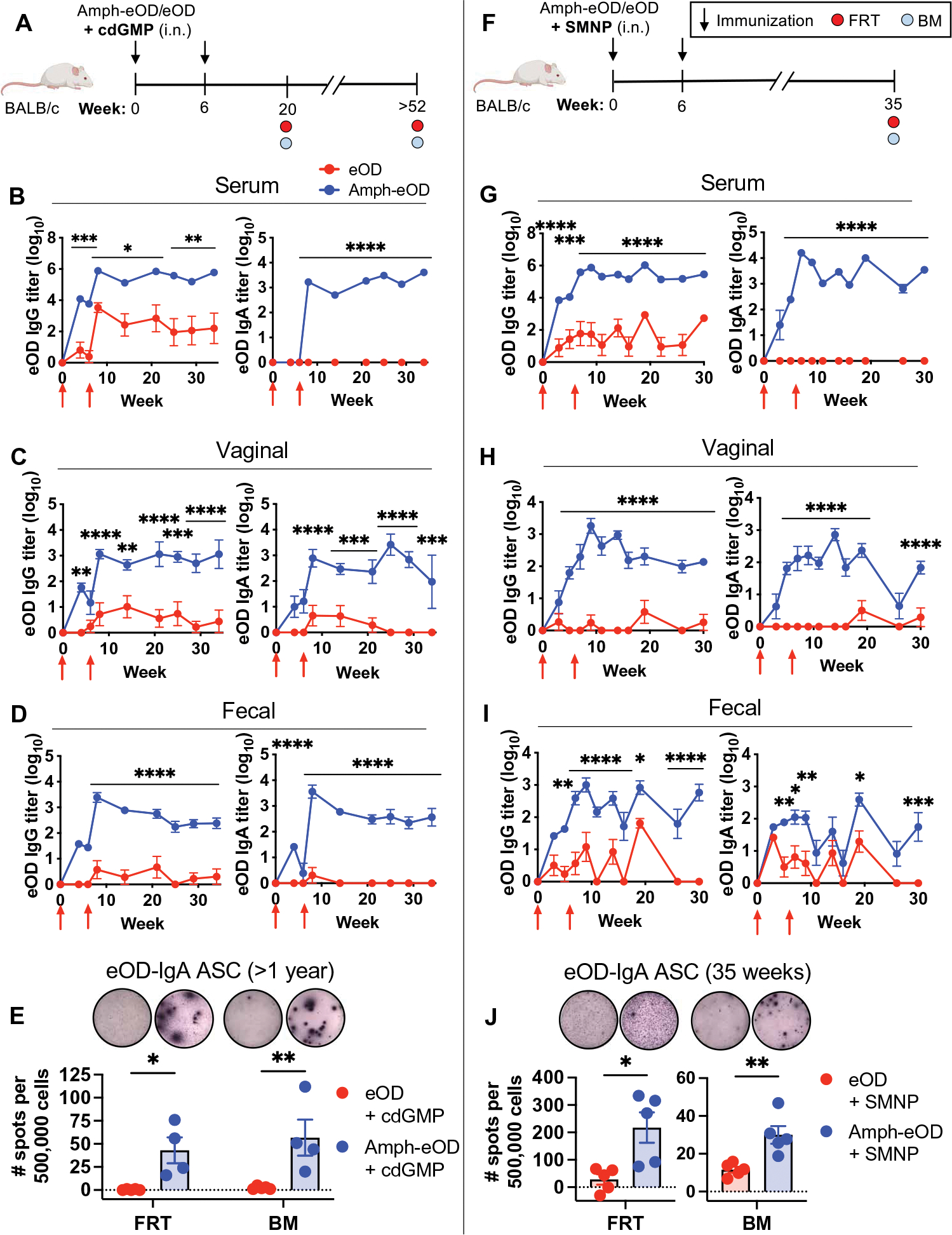
Amph-protein conjugates elicit enhanced systemic and mucosal immune responses after intranasal vaccination. (**A** to **E**) BALB/c mice (*n* = 5 animals per group) were immunized intranasally with 5 μg of eOD or amph-eOD mixed with 25 μg of cdGMP adjuvant and boosted 6 weeks later with the same formulations. (A) A schematic illustrating the experimental timeline is shown. (B to D) IgG and IgA titers were measured in the serum (B), vaginal wash (C), and feces (D). Red arrows indicate vaccination. (E) FRT and BM eOD-specific IgA ASCs were assessed by ELISPOT greater than 1 year after immunization. Data shown are from one representative of two independent experiments. (**F** to **J**) BALB/c mice (*n* = 5 animals per group) were immunized with 5 μg of eOD or amph-eOD mixed with 5 μg of SMNP adjuvant and boosted 6 weeks later with the same formulations. (F) A schematic illustrating the experimental timeline is shown. (G to I) IgG and IgA titers were measured in the serum (G), vaginal wash (H), and feces (I). (J) FRT and BM eOD-specific IgA ASCs were assessed by ELISPOT 35 weeks after immunization. Data shown are from one representative of two independent experiments (minus naïve background). Statistical significance in (E) and (J) was determined by unpaired *t* test, and that in (B) to (D) and (G) to (I) was determined by ordinary two-way ANOVA followed by Sidak’s post hoc test, comparing eOD to amph-eOD at each time point. *P < 0.05, **P < 0.01, ***P < 0.001, and ****P < 0.0001. All data show means ± SEM.

We next evaluated output antibody responses elicited by intranasal amphiphile or soluble protein immunization both systemically and at distal mucosal sites relevant for HIV transmission such as the rectal and genitourinary mucosa. First, we carried out studies combining eOD with the cyclic dinucleotide (CDN), cyclic di-guanosine monophosphate (cdGMP; Fig. 4A). CDNs activate the innate immune sensor stimulator of interferon genes (STING) and have been previously reported to be an effective mucosal vaccine adjuvant in mice (43–45). Intranasal immunization with amph-eOD and cdGMP induced very high serum IgG and IgA responses, with end point antigen-specific serum IgG titers of about 10^6^ and IgA titers of 10^3^ to 10^4^ that were sustained over 35 weeks (Fig. 4B). Amph-vaccination increased IgG responses over unmodified eOD by more than 2 logs and primed strong serum IgA responses that were completely absent after soluble protein immunization (Fig. 4B). Amph-eOD also induced sustained mucosal IgG and IgA responses in the vaginal tract (Fig. 4C) and rectal mucosa (Fig. 4D), where soluble eOD immunization again elicited only weak to undetectable responses. We also directly compared intranasal versus parenteral (subcutaneous) vaccination with amph-eOD. Subcutaneous immunization with amph-eOD elicited potent systemic IgG titers in blood but failed to prime mucosal responses (fig. S7). Next, cohorts of mice were euthanized at different time points, and the female reproductive tract (FRT) and bone marrow (BM) were isolated and analyzed by antibody-secreting cell (ASC) enzyme-linked immunosorbent spot (ELISPOT) assay to identify long-lived plasma cells. Amph-eOD immunization led to high numbers of both eOD-specific IgA and IgG plasma cells in the FRT and BM 20 weeks after immunization (fig. S8, A and B). Furthermore, more than 1 year after immunization, mice immunized with amph-eOD retained significantly higher populations of eOD-specific IgA plasma cells resident in the FRT (*P* < 0.05) and BM (*P* < 0.01) than eOD-immunized mice (Fig. 4E).

CDNs are in clinical trials as immunostimulators for cancer therapy but have yet to be used with vaccines in humans. We thus next carried out a similar study using an immune stimulating complex (ISCOM)–like saponin adjuvant called saponin monophosphoryl lipid A (MPLA) nanoparticles (SMNPs) (46), which has a nanoparticle structure and composition similar to the Matrix M adjuvant in advanced clinical testing for SARS-CoV-2 vaccines by Novavax (Fig. 4F) (47). Similar to CDNs, ISCOM-based adjuvants have been shown to be effective intranasal adjuvants in preclinical studies (48, 49). Similar to the findings with cdGMP, intranasal immunization with amph-eOD and SMNP induced notable serum eOD-specific IgG and IgA titers of about 10^6^ and 10^4^, respectively, exceeding those induced by unmodified eOD at all time points before and after boost (Fig. 4G). Amph-eOD/SMNP immunization also induced robust long-term mucosal IgG and IgA responses in the vaginal tract (Fig. 4H) and rectal mucosa (Fig. 4I), with amph-eOD post-boost titers consistently 10^3^-fold higher than those from eOD in the vaginal mucosa and 10- to 100-fold higher in fecal samples. After 35 weeks, the FRT and BM were analyzed by ASC ELISPOT, again showing significantly elevated numbers of eOD-specific IgA plasma cells in the FRT (*P* < 0.05) and BM (*P* < 0.01) of mice immunized with amph-eOD compared to eOD (Fig. 4J). With both cdGMP and SMNP adjuvants, the population of IgA plasma cells established in the FRT was similar or greater in magnitude to that in the BM (Fig. 4, E and J). Together, these studies indicate that intranasal immunization with amph-conjugated antigen can promote robust long-term systemic and mucosal antigen-specific humoral immunity in mice with multiple adjuvants.

Recently, some concerns have arisen from clinical studies of the SARS-CoV-2 mRNA vaccines regarding the possibility of antibody responses against PEG included in vaccine formulations, which might induce allergic reactions in human volunteers (50, 51). We thus analyzed serum samples from the studies above using saponin or cdGMP adjuvants for the presence of anti-PEG IgG. We observed that, despite the use of strong adjuvants, anti-PEG responses elicited by amph-eOD were barely above background (fig. S8C).

### High titers of nAbs against SARS-CoV-2 can be induced in the respiratory mucosa by amph-vaccination

**Fig. 5.**
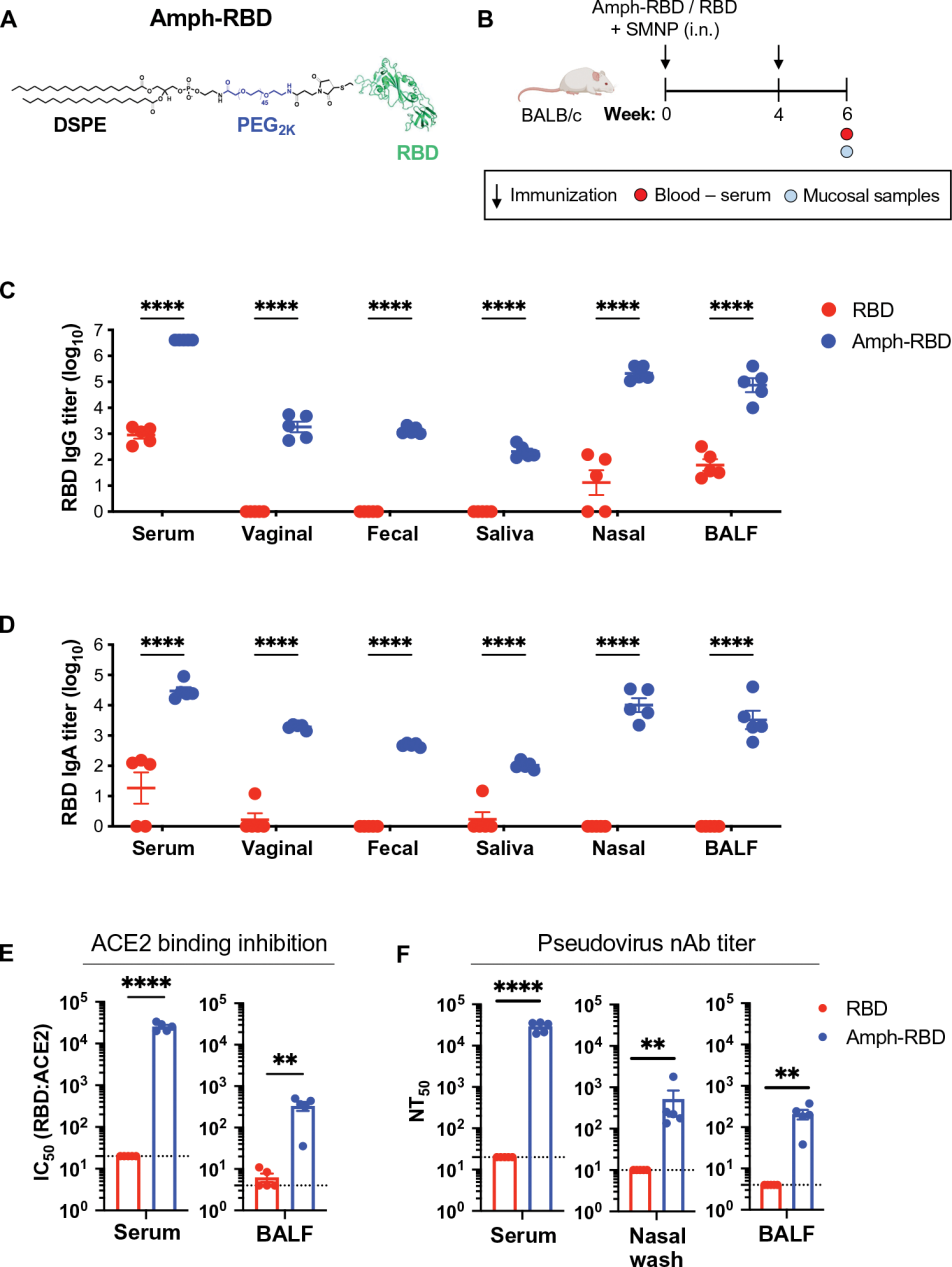
Intranasal vaccination with an amph-RBD conjugate elicits enhanced systemic and mucosal neutralizing antibody responses to SARS-CoV-2 immunogens. (**A**) A schematic of amph-RBD structure is shown. (**B**) BALB/c mice (*n* = 5 animals per group) were immunized intranasally with 5 μg of RBD or amph-RBD mixed with 5 μg of SMNP adjuvant and boosted 4 weeks later with the same formulations. (**C**) IgG and (**D**) IgA titers in the serum, vaginal wash, fecal wash, saliva, nasal wash, and bronchoalveolar lavage fluid (BALF) were measured at 6 weeks after immunization. (**E**) ACE2:RBD binding inhibition (IC_50_) was measured for antibodies in serum and BALF at 6 weeks after immunization. (**F**) Pseudovirus neutralizing antibody (nAb) titers (NT_50_) were measured in the serum, nasal wash, and BALF at 6 weeks after immunization. Dotted lines in (E) and (F) represent the limit of quantitation. Data shown are from one representative of two independent experiments. Statistical significance in (C) and (D) was determined by two-way ANOVA followed by Sidak’s post hoc test, and that in (E) and (F) was determined by unpaired *t* test. **P < 0.01 and ****P < 0.0001. All data are presented as means ± SEM.

eOD is a germ line–targeting immunogen designed to initiate priming of human B cells with the capacity to produce broadly neutralizing antibodies similar to the CD4 binding site broadly neutralizing antibody, VRC01 (38–41); however, this immunogen cannot induce nAb responses in WT mice due to genetic differences in the complementarity-determining region 3 genes encoding murine versus human antibodies. Furthermore, responses induced in the local respiratory mucosa by intranasal immunization are not relevant for protection from HIV. These considerations motivated us to test the utility of amph-conjugation in the setting of vaccines for SARS-CoV-2 because WT mice readily produce nAbs against this virus, and nAb responses in the nasal passages and airways are highly relevant for protection (52–54). We chose the RBD of the SARS-CoV-2 spike protein as the target antigen to incorporate into the amphiphile platform because it is the target of most human nAbs (55). Soluble RBD protein is known to be poorly immunogenic (56, 57); we set out to determine whether amphiphile conjugation of RBD would enhance its immunogenicity and promote protective systemic and respiratory mucosal antibody responses in tandem. To this end, we used an engineered RBD immunogen that we recently developed, which is expressed in *Pichia pastoris* in much higher quantities and exhibits substantially greater stability than the WT RBD sequence (58). Modifying the RBD immunogen with an N-terminal cysteine did not affect its production, stability, or antigenicity profile (fig. S9, A and B) and allowed us to easily conjugate the protein with maleimide-functionalized PEG_2K_-DSPE (Fig. 5A). Similar to amph-eOD, conjugated amph-RBD formed roughly 35-nm-diameter micelles in aqueous solution, facilitating purification from unreacted RBD (5 nm) by size exclusion chromatography (fig. S9, C and D).

To assess the immunogenicity of amph-RBD, BALB/c mice were immunized intranasally with amph-RBD or RBD combined with SMNP adjuvant at 0 and 4 weeks; at week 6, serum and mucosal samples were collected and assayed for RBD-specific IgG and IgA titers and pseudovirus neutralization (Fig. 5B). Amph-RBD outperformed soluble RBD for eliciting antigen-specific serum and mucosal IgG and IgA responses (Fig. 5, C and D). Serum IgG and IgA titers were three orders of magnitude greater for amph-RBD versus RBD, and amph-RBD elicited potent IgG and IgA responses in nasal washes and bronchiolar lavage fluid (BALF), where soluble RBD immunization elicited weak or no responses (Fig. 5, C and D). An angiotensin-converting enzyme 2 (ACE2)–RBD binding inhibition assay revealed a half maximal inhibitory concentration (IC_50_) for blocking ACE2 binding by RBD of about 25,000 in the serum and about 300 in the BALF from amph-RBD–immunized mice (Fig. 5E and fig. S9, E and F). Last, analysis of SARS-CoV-2 pseudovirus neutralization revealed serum nAbs at titers of about 30,000, and mean nasal and BAL nAb titers of about 500 and about 200, respectively (Fig. 5F). In contrast, intranasal immunization with soluble RBD elicited no detectable neutralizing response in any compartment (Fig. 5F). Thus, intranasal amph-RBD vaccination markedly enhances the induction of nAb responses at mucosal portals of entry for the SARS-CoV-2 virus.

### Amph-conjugated vaccines exhibit enhanced immunogenicity in nonhuman primates

**Fig. 6.**
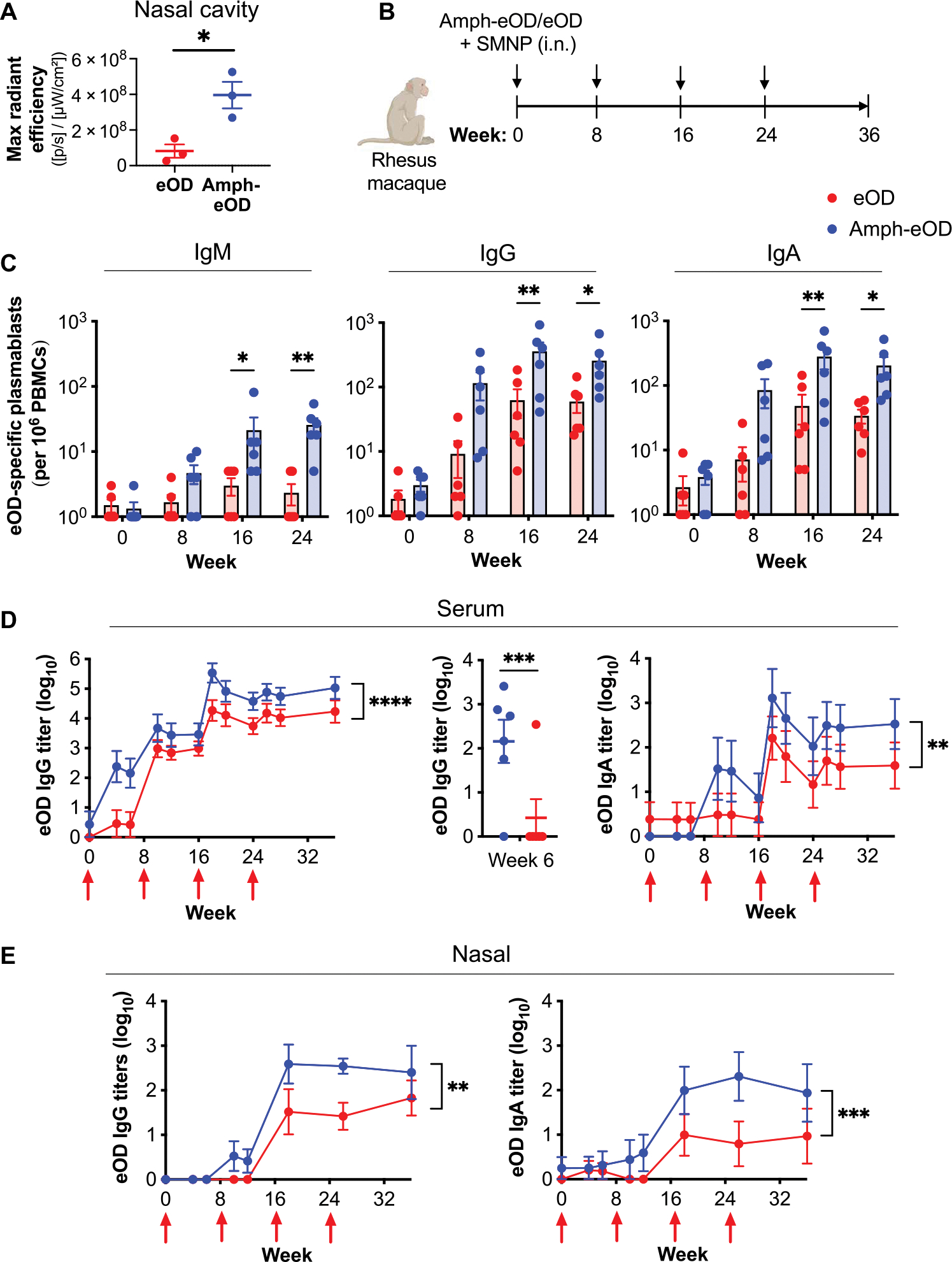
Intranasal immunization with amph-protein conjugates leads to improved humoral immune responses in nonhuman primates. (**A**) Rhesus macaques (*n* = 3 animals per group) were immunized intranasally with 100 μg of AF647-eOD or AF647-amph-eOD mixed with 375 μg of SMNP adjuvant. Shown is quantified fluorescence signal of vaccine immunogens in the nasal cavity after 24 hours by IVIS imaging. Statistical significance was determined by unpaired *t* test. (**B**) Rhesus macaques (*n* = 6 animals per group) were immunized intranasally with 100 μg of eOD or amph-eOD mixed with 375 μg of SMNP adjuvant and boosted at 8, 16, and 24 weeks with the same formulations. (**C**) Frequencies of antigen-specific IgM, IgG, and IgA secreting plasmablasts of total PBMCs were measured by ELISPOT. (**D**) IgG and IgA titers in the serum were quantified over time; statistical significance shows an overall comparison across all time points. Individual IgG titers at 6 weeks are shown in the middle plot. (**E**) IgG and IgA titers in the nasal wash were quantified over time; statistical significance shows an overall comparison across all time points. Statistical significance in (C) to (E) was determined by two-way ANOVA. *P < 0.05, **P < 0.01, ***P < 0.001, and ****P < 0.0001. All data are presented as means ± SEM.

The systemic and mucosal antibody responses elicited by amph-conjugate vaccines in mice were compelling, but many vaccine technologies that are effective in small animals fail to translate well to larger animals and humans. Thus, we next sought to evaluate whether amph-conjugates would also be effective in nonhuman primates using the eOD immunogen. We first evaluated trafficking of the amphiphile vaccine versus soluble protein after intranasal immunization in rhesus macaques. AF647-labeled amph-eOD or soluble eOD was administered intranasally with SMNP adjuvant; after 24 hours, the tonsils, adenoids, cervical lymph nodes, axillary lymph nodes, and nasal tissue including turbinates were collected and evaluated by IVIS imaging for fluorescence signal from the labeled immunogens. Similar to our observations in mice, amph-eOD was detected in the nasal tissue at significantly higher (*P* < 0.05) concentrations than eOD (Fig. 6A).

To assess vaccine immunogenicity, rhesus macaques were immunized intranasally with amph-eOD or eOD combined with SMNP at 0, 8, 16, and 24 weeks (Fig. 6B). Peripheral blood mononuclear cells (PBMCs) were collected 5 days after each immunization to assay plasmablast responses by ASC ELISPOT. Amph-eOD induced significantly higher eOD-specific IgM (*P* < 0.05 and *P* < 0.01), IgG (*P* < 0.01 and *P* < 0.05), and IgA (*P* < 0.01 and *P* < 0.05) plasmablast responses after the second and third boosts, respectively, quantified as total number of antigen-specific plasmablasts or as a percentage of total plasmablasts (Fig. 6C and fig. S10, A and B). In the serum, amph-eOD intranasal immunization seroconverted all animals after a single dose, whereas serum IgG titers primed by soluble eOD were near baseline until the first boost was administered (Fig. 6D). Antigen-specific serum IgG and IgA titers were consistently about 10-fold higher in macaques immunized with amph-eOD compared to eOD even after repeated boosting (Fig. 6D). In the nasal mucosa, IgG and IgA were about 1 log higher in macaques immunized with amph-eOD compared to eOD at weeks 18 and 26 and were sustained after boosting (Fig. 6E). Distinct from the findings in mice, amph-eOD elicited sporadic vaginal and rectal IgG and IgA responses. Although overall vaginal IgG, vaginal IgA, and rectal IgG from amph-eOD were significantly greater than eOD (*P* < 0.001, *P* < 0.01, and *P* < 0.0001, respectively), these responses were not consistently sustained throughout the study (fig. S10, C and D). Together, these data in the closest available animal model to humans suggest that amph-conjugated intranasal immunization is a promising strategy for enhancing both systemic and mucosal immunity to subunit vaccines.

## DISCUSSION

We previously demonstrated that linking peptide antigens to amphiphilic lipid tails promotes albumin-mediated transport into lymphatics after parenteral injection, thereby enhancing antigen-specific T cell responses that are critical for cancer immunity (25, 27, 29). Here, we demonstrate that this strategy can be used with much larger protein immunogens relevant for humoral immunity and that albumin hitchhiking can be applied to enhance intranasal delivery of immunogens by exploiting another natural transport mechanism of endogenous albumin: its capacity to be transcytosed across the mucosal epithelium by the FcRn (31, 32). Amph-proteins showed prolonged residence in the nasal tissue after intranasal administration in both mice and nonhuman primates. In mice, we demonstrated that this persistence was linked to increased transport across the mucosal barrier and greater uptake in the NALT. The NALT is a secondary lymphoid organ located on the dorsal side of the soft palate underlying the nasal passage in rodents, analogous to the Waldeyer’s ring in primates and humans (16). In mice, the NALT consists of focal aggregates, whereas in primates the Waldeyer’s ring is more abundant, consisting of tonsils and adenoids (15, 59). The NALT, tonsils, and adenoids all serve as key sites for initiation and orchestration of local mucosal antigen-specific immune responses (3, 60, 61). We found substantial increases in GC B cell and Tfh cell responses in the NALT after intranasal immunization with amph-conjugated immunogens when compared to free proteins. This increased antigen delivery and local immune priming correlated with enhanced systemic IgG and IgA responses, as well as mucosal antibody responses, in both mice and nonhuman primates. Amph-modification of protein immunogens enabled intranasal immunizations to elicit strong serum IgG responses in conjunction with robust mucosal IgA responses. This is of great interest, as many infectious diseases such as SARS-CoV-2, influenza, rotavirus, and cholera are thought to require a combination of mucosal IgA and serum IgG antibodies for optimal protection (1–7). Thus, the ability to activate both systemic IgG and mucosal IgA is likely to be of value in developing vaccines for diverse pathogens.

Amph-proteins overcome a major obstacle to mucosal vaccine development: delivery of antigens across the mucus and epithelial barrier to the underlying mucosal immune compartment (18, 19). In addition to efficient mucociliary clearance mechanisms, mucosal surfaces are lined with epithelial monolayers formed by intercellular tight junctions that prevent macromolecular uptake by diffusion (20). Thus, transport of molecules across the nasal mucosal epithelium is thought to be restricted to active transport of small soluble proteins by goblet cells (22, 62), and transport of larger inert particulates by differentiated microfold cells (M cells). Similar to Peyer’s patches in the gut, M cells are also found lining the nasal cavity, both in the turbinate epithelium and in follicle-associated epithelium overlaying the NALT where they sit atop subepithelial domes (SEDs) of organized mucosal lymphoid tissue and act as “antigen delivery cells” (16, 63, 64). Here, M cells acquire antigen from the nasal mucosal lumen; transcytose it across the submucosal epithelium; and then hand off antigen to underlying dendritic cells, macrophages, B cells, and other APCs in the SED. After intranasal administration, we observed a substantial amount of amph-eOD concentrated in the nasal turbinates, which may have allowed for M cell capture and transcytosis to serve as another mechanism for intranasal amph-eOD uptake (62, 65). However, FcRn-expressing columnar epithelial cells are much more abundant than M cells in the respiratory mucosa (62). This, in combination with our data showing a clear dependence of amph-eOD uptake and immune responses on FcRn, indicates that FcRn-mediated transcytosis is a more efficient pathway for antigen delivery in the nasal mucosa. Albumin-bound amph-antigens transcytosed by respiratory epithelial cells would be released at the basolateral surface, where they can then be taken up by underlying APCs. APCs such as macrophages, dendritic cells, and B cells, where we observed the highest amph-eOD uptake, also express high concentrations of FcRn (66).

Recognized for its role in recycling and extending the half-lives of IgG and albumin, FcRn is increasingly targeted as a means to alter drug delivery and drug pharmacokinetics (30, 31, 34). To date, the focus has largely been on developing engineered therapeutic monoclonal antibodies (such as Fc-fusions) with altered FcRn binding affinities or drug-albumin fusions; these modifications extend serum half-life by exploiting FcRn-mediated recycling in the blood and increasing overall molecular weight to reduce the rate of kidney clearance. More recently, the FcRn transcytosis pathway has been explored for noninvasive protein delivery through FcRn-mediated transcytosis (42, 67–69). For example, Pridgen *et al.* (67) observed about 10-fold higher uptake across the intestinal epithelium with FcRn-targeted nanoparticles versus nontargeted nanoparticles as a means to orally deliver encapsulated insulin across the intestinal mucosa in mice, whereas Bern *et al.* (42) found that an engineered albumin-protein fusion with improved FcRn binding exhibited enhanced uptake across the nasal epithelium and increased serum half-life in mice.

More directly relevant to the present study, Roopenian and Zhu demonstrated that fusions of protein antigens with antibody Fc domains can enhance intranasal vaccination against HSV-2 (70) and HIV Gag (71). These antigen-Fc fusions enhanced systemic antibody and T cell responses to intranasal immunization and mucosal antibody in BALF and vaginal fluid, but to our knowledge, this approach has not been evaluated for efficacy in large animal models. An important distinction between approaches solely leveraging FcRn interactions and the amph-vaccine approach studied here is that Fc or albumin fusions administered to airway surfaces not only are delivered to the local mucosal lymphoid tissues but also reach the systemic circulation and thereafter exhibit circulation times in the blood seen for antibodies and albumin; this has motivated the use of Fc and albumin fusions for delivery of systemic therapeutics such as erythropoietin (68, 69). However, such broad distribution is problematic for vaccines. Vaccine adjuvants by design provide very localized inflammatory cues to avoid systemic toxicity, but if antigens coadministered with these adjuvants do not also remain localized, then a competing tolerogenic response can develop in uninflamed distal lymphoid tissues, such as lymph nodes and spleen (72). By contrast, the lipid tail of amphiphile conjugates promotes cell membrane interactions that prevent systemic dissemination of these conjugates. We previously demonstrated that amph-peptides administered subcutaneously accumulate efficiently in draining lymph nodes but do not reach the systemic compartments (26). Here, we have shown similar localized stimulation of immune responses after intranasal administration of amph-proteins, which activated responses in the NALT but did not substantially reach even the nearby draining cervical lymph nodes or accumulate in tissues such as the spleen, liver, and intestines, indicating negligible systemic distribution. The membrane insertion property of amph-proteins promotes this localization but does not lead to chronic antigen exposure because PEG-lipids are cleared from cell surfaces over the course of a few days (26).

Development of an amph-RBD SARS-CoV-2 vaccine demonstrated the ability of this amph-protein vaccine platform to induce functional nAb responses at mucosal sites of respiratory pathogen entry. Clinical studies have shown that mucosal IgA is a strong correlate of protection against SARS-CoV-2 (6, 13, 14), but to date, most SARS-CoV-2 vaccines have not focused on targeting mucosal tissues and few have been shown to induce functional neutralization at mucosal sites (54, 73, 74). Amph-RBD immunization induced notable IgG and IgA antibody responses, including nAbs, in both serum and the upper and lower respiratory mucosa in mice. Thus, intranasal amph-RBD vaccination may be a promising approach for eliciting mucosal protection against SARS-CoV-2 infection. In addition, needle-free mucosal vaccination provides practical advantages over parenteral vaccination in cases where mass vaccination is needed, such as the current global COVID-19 pandemic. Easier administration, delivery that does not require personnel with medical training, better compliance, and avoiding risks of spreading blood-borne infections through needle contamination can all lead to better vaccination rates (3). From a translational perspective, we expect that amph-protein vaccines are readily manufacturable. Monodisperse functionalized PEG-lipids are available for clinical manufacturing, and a peptide-PEG-DSPE amphiphile is currently in early stage clinical testing as a cancer vaccine (NCT04853017).

A limitation of these studies is the inherent challenge of immunological differences between animal models and humans. In mice, amph-protein immunization not only elicited robust local mucosal antibody responses but also stimulated long-lived, high titer IgG and IgA at distal vaginal and rectal mucosal sites, accompanied by generation of resident ASCs. By contrast, intranasal amph-protein immunization in nonhuman primates elicited enhanced systemic and nasal IgG and IgA responses compared to soluble protein administration, but distal mucosal responses in the vaginal tract and rectum were not sustained. Such “common mucosal immunity” has been reported in other small studies in macaques (1, 11, 75–77) and humans. For example, intranasal immunization with the strong mucosal adjuvant cholera toxin B in humans elicited antibody responses in urine or vaginal secretions (78, 79). Concerns regarding the safety of adjuvants for intranasal vaccination remain a challenge, and cholera toxin B has not advanced as an intranasal adjuvant because of its associated risk of triggering Bell’s palsy (77). However, these data suggest that with appropriate adjuvants, distal mucosal responses can be elicited in humans. Despite this limitation, the strong systemic and local mucosal antibody priming observed here in rhesus macaques after intranasal amph-protein administration combined with the saponin adjuvant SMNP, an adjuvant currently in GMP development for a first-in-humans clinical trial, suggest promise for this approach to be valuable for human vaccines. Together, these results suggest that using amphiphile-protein vaccines to deliver antigen across the mucosal epithelium presents a promising and simple strategy to promote mucosal immunity against HIV, SARS-CoV-2, and other infectious diseases.

## MATERIALS AND METHODS

### Study design

The major objective of this study was to evaluate the effect of modifying protein antigens with an amphiphilic PEG-lipid tail on systemic and mucosal immune responses elicited by intranasal vaccination in small and large animal models and to define mechanisms of action underlying the action of these modified immunogens. Mice and nonhuman primates were immunized with clinically relevant subunit protein immunogens combined with saponin or alternate adjuvants, and early local responses (antigen uptake, T cell priming, and GC induction) and later event (serum and mucosal antibody, plasmablast, and plasma cell) responses were assessed over time. Murine immunization study sampling and ELISAs were carried out blinded. Group sizes for immunogenicity studies were selected on the basis of effect sizes seen in pilot studies aiming for 80% power to detect a difference of 20% or more between experimental groups. For mechanistic studies, we used fluorescently labeled proteins enabling immunogen trafficking in tissues and genetic knockout mouse models to dissect key pathways in the immune response. For exclusion criteria, flow cytometry data were omitted if the sample’s total cell counts were less than 30,000.

### HIV eOD and SARS-CoV-2 RBD immunogen synthesis and characterization

eOD-GT8 gp120 protein was synthesized as previously described (38, 80). The eOD protein, with a free N-terminal cysteine and C-terminal PADRE universal helper T cell epitope (AKFVAAWTLKAAA), was expressed in Expi293F human embryonic kidney (HEK) cells (Thermo Fisher Scientific). The eOD protein was purified on a nickel affinity column followed by size exclusion chromatography on a Superdex 75 10/300 column (GE Healthcare).

An engineered RBD protein (“RBD-L452K-F490W”) was produced in *Komagataella phaffii* (*P. pastoris*). This strain was cultivated in 200 ml of flask culture, and secreted protein was purified as previously described (58). For amphiphile conjugation, the RBD was genetically modified to include an N-terminal cysteine residue.

Immunogens were administered in vivo with adjuvant. The STING agonist adjuvant bis-(3′-5′)-cdGMP was purchased from InvivoGen. SMNP adjuvant was synthesized as previously described (81).

### Murine strains

All procedures were approved by the Massachusetts Institute of Technology Institutional Animal Care and Use Committee (IACUC). Procedures followed local, state, and federal regulations (protocol 0720-070-23). Immunization studies were carried out using age-matched 8- to 10-week-old female BALB/cJ mice (strain 000651), C57BL/6J mice (strain 000664), or FcRn^−/−^ mice on a C57BL/6J background (strain 003982) purchased from the Jackson Laboratory.

### Mouse immunizations and blood collection

BALB/c mice were immunized intranasally by administering vaccines in 20 μl of phosphate-buffered saline (PBS; 10 μl per nare with 30- to 60-s interval between nares) with the mouse anesthetized in the supine position. Animals were primed on day 0 and boosted on day 28 or 42 with a 5-μg dose of eOD or RBD combined with 25 μg of cdGMP or 5 μg of SMNP adjuvant, as indicated. For longitudinal immune monitoring, blood and mucosal samples were collected bi- or triweekly for ELISA or pseudovirus neutralization test (PVNT) antibody analysis, as indicated. Blood was collected by cheek or retro-orbital bleed; serum was isolated using serum separator tubes and centrifuged at 10,000*g* for 5 min to collect supernatant.

### Macaque immunization study and sample collection

Female rhesus macaques were immunized intranasally at weeks 0, 8, 16, and 24 with 100 μg of amph-eOD or eOD mixed with 375 μg of SMNP, as described above. For longitudinal immune monitoring, PBMCs were collected by venipuncture from the femoral vein and then Ficoll-separated and cryopreserved except for those used freshly for plasmablast ELISPOT assay. Serum samples were stored at −80°C until ELISA analysis. Mucosal samples were collected by using Merocel sponges and processed as previously described (82) and stored at −80°C until analysis.

### Statistical analysis

Raw, individual-level data are presented in data files S1 and S2. Statistics were analyzed using GraphPad Prism software. For comparison of more than two groups, one- or two-way analysis of variance (ANOVA) was performed with α = 0.05, followed by Tukey’s or Sidak’s post hoc test as indicated. For comparison of two groups, two-tailed unpaired *t* test was performed with α = 0.05. For comparison of groups not following a normal distribution, a nonparametric Mann-Whitney *U* test was performed followed by a Holm-Sidak correction with α = 0.05. Statistical significance in amphiphile membrane insertion experiments was determined using simple linear regression evaluating the dependence of AF647 or VRC01 mean fluorescence intensity on eOD concentration to determine significant nonzero slope. ACE2:RBD binding inhibition (IC_50_) was determined using sigmoidal four parameter logistic (4PL) nonlinear regression. All graphs represent means ± SEM unless otherwise noted. Statistical significance is marked as *P < 0.05, **P < 0.01, ***P < 0.001, and ****P < 0.0001.

## Supplementary Material

Supplementary Materials

MDAR Reproducibility checklist

Data file S2

Data file S1

## Data Availability

All data associated with this study are in the paper or the Supplementary Materials.
