# Supplementary material for: Intranasal vaccination with lipid-conjugated immunogens promotes antigen transmucosal uptake to drive mucosal and systemic immunity
Source: Sci Transl Med. Author manuscript; Available in PMC 2023 Jan 12. (PMC9835395; doi:10.1126/scitranslmed.abn1413)
Supplement: Supplementary Materials [file NIHMS1853909-supplement-Supplementary_Materials.pdf]

Science Translational Medicine
AAAS

Supplementary Materials for

Intranasal vaccination with lipid-conjugated immunogens promotes antigen transmucosal uptake to drive mucosal and systemic immunity

Brittany L. Hartwell *et al.*

Corresponding author: Darrell J. Irvine, djirvine@mit.edu

Sci. Transl. Med. **14**, eabn1413 (2022)
DOI: 10.1126/scitranslmed.abn1413

The PDF file includes:

Materials and Methods
Figs. S1 to S10
Legends for data files S1 and S2
References (*83–87*)

Other Supplementary Material for this manuscript includes the following:

MDAR Reproducibility Checklist
Data files S1 and S2

Materials and Methods

Amphiphile conjugation and labeling

HIV env gp120 engineered outer domain (eOD) and SARS-CoV-2 spike receptor binding domain (RBD) protein antigens with N-terminal cysteines (≥1mg/ml) were first reduced with 10 molar equivalents of tris(2-carboxyethyl)phosphine (TCEP) for 15 minutes at 25°C. TCEP was removed through centrifugal filtration using 10 kDa molecular weight cutoff (MWCO) Amicon spin filters while washing the protein three times with phosphate-buffered saline (PBS). Proteins (1 to 5mg/ml) were then reacted with 4 equivalents of dried DSPE-PEG2K-maleimide (1,2-distearoyl-sn-glycero-3-phosphoethanolamine-N-[maleimide(polyethylene glycol)-2000]) (Avanti Polar Lipids) in PBS for 2 hours at 25°C with intermittent vortexing, followed by gentle mixing for 18 hours at 4°C.

Protein amphiphiles were purified by size exclusion chromatography using a Sepharose CL6B (Sigma-Aldrich) gravity column eluted with PBS. The conjugated protein-amphiphile micelle and unconjugated protein peaks were detected by tryptophan fluorescence (excitation: 280nm/emission: 340 nm). Micelle peak fractions were pooled, concentrated through centrifugal filtration using 10 kDa MWCO Amicon spin filters, and quantified by UV-Vis spectrophotometry (Nanodrop One, Thermo Fisher Scientific). Particle size was characterized by dynamic light scattering (Zetasizer Nano, Malvern).

Labeled eOD protein and eOD protein amphiphile conjugate (amph-eOD) were prepared using Alexa Fluor (AF) 647 N-hydroxysuccinimide (NHS) ester (Thermo Fisher Scientific) by reaction of fluorophore with eOD or amph-eOD (≥1mg/ml) in 0.1M sodium bicarbonate buffer for 1 hour at 25°C, per the manufacturer instructions. VRC01 was synthesized as previously described *(88)*; labeled VRC01 was prepared using Pierce NHS-Rhodamine (Thermo Fisher Scientific) by reaction of the fluorophore with human VRC01 (≥1mg/ml) in PBS for 1 hour at 25°C, per the manufacturer instructions. Labeled proteins were purified by centrifugal filtration using 10kDa Amicon spin filters; degree of labeling (DOL) was characterized by UV-Vis spectrophotometry and confirmed to be ≥1.0.

Albumin binding: affinity chromatography

Albumin binding of conjugates was evaluated using albumin-immobilized agarose affinity chromatography as previously described *(25)*. Pierce NHS-activated agarose resin (Thermo Fisher Scientific) was functionalized with albumin by adding 26.4 mg bovine serum albumin (BSA) in 4.4 ml PBS directly to 330 mg agarose, per the manufacturer instructions. The resin reaction was mixed for 1 hour at 25°C followed by 4°C overnight, then quenched with 1M Tris-HCl (pH 8.0) followed by extensive washing with PBS. Next, AF647-labeled eOD or amph-eOD was applied to the albumin-functionalized resin (0.3 µM final concentration in 2 ml column volume) and incubated with end-over-end mixing for 2 hours at 37°C. Eluent was collected following column centrifugation at 1000*xg* for 2 minutes. The amount of protein or amphiphile conjugate retained in the column was determined by measuring AF647 fluorescence (640/670 nm) of the eluent versus starting sample on a fluorescent plate reader and normalizing by DOL.

Membrane insertion in splenocytes

Amphiphile insertion into cell membranes was evaluated in vitro in murine splenocytes isolated from naïve BALB/cJ mice (The Jackson Laboratory, strain 000651). Single cell suspensions were

incubated at $5x10^6$ cells per ml ($1x10^6$ cells per well in a 96-well plate) in cRPMI (RPMI-1640 + 10% fetal bovine serum (FBS) + 1% penicillin/streptomycin) containing 25, 100, or 250 nM AF647-eOD or AF647-amph-eOD for 1 hour at 37°C. Cells were washed once with PBS, stained with Live/Dead Aqua (Invitrogen) at 1:1000 in 100 µl PBS for 15 minutes at 25°C, washed once in FACS buffer (PBS+1% BSA), then stained with Rhodamine-VRC01 at 1.0 µg/10^6 cells in 100 µl FACS buffer for 30 minutes at 4°C. Cells were then washed twice, fixed with 2% paraformaldehyde, and stored at 4°C until flow cytometry analysis on a BD LSR Fortessa.

Albumin-Neonatal Fc receptor (FcRn) binding measurements

To measure FcRn binding, 96-well enzyme-linked immunosorbent assay (ELISA) plates (Corning, #3690) were coated with 5.0 µg/ml streptavidin in phosphate-buffered saline (PBS) and incubated for 4 hours at 25°C, blocked for 18 hours at 4°C with 1% casein in PBS (G-biosciences, 786-194), then washed three times with PBS+0.05% Tween 20 (pH 5.5). Biotinylated human FcRn (ACRO Biosystems, FCMH82W4) was added at 5 µg/ml in 1% casein in PBS (pH 5.5) and incubated for 2 hours at 25°C prior to washing. Human albumin (Sigma, A3782, serially diluted 5–0 µg/ml) was pre-incubated for 2 hours at 25°C with fluorescein isothiocyanate (FITC)-labeled 1,2-distearoyl-sn-glycero-3-phosphoethanolamine-N-[poly(ethylene glycol)-2000] (DSPE-PEG_{2K}-FITC, Creative PEGworks, PLS-9927, serially diluted 10–0 µM) in 1% casein in PBS (pH 5.5), then added to the FcRn-coated plates and incubated for an additional 2 hours at 25°C. Goat anti-Human Albumin Antibody, horseradish peroxidase (HRP)-Conjugated (Bethyl Laboratories, A80-129P), diluted 1:3000 in 1% casein in PBS, was added and incubated for 30 minutes at 25°C. Plates were washed three times before adding tetramethylbenzidine (TMB) substrate (Thermo Fisher Scientific, 34028) followed by 2 N H_2SO_4 as a stop solution. Absorbance was measured at 450 nm.

IVIS trafficking in murine tissues

In vivo trafficking of AF647-labeled amph-eOD and eOD was evaluated following intranasal administration using In Vivo Imaging System (IVIS) fluorescence imaging (Perkin Elmer). Mice were fed an alfalfa-free diet (AIN-93M, Bio-Serv) for the duration of the study, starting 3 days before immunization, to eliminate background auto-fluorescence in the gut. BALB/c mice were immunized intranasally with 5 µg AF647-amph-eOD or AF647-eOD combined with 5 µg saponin monophosphoryl lipid A (MPLA) nanoparticle (SMNP) adjuvant, and compared to a naïve control. Intranasal immunizations were administered dropwise in 20 µl PBS (10 µl per nare with 30 to 60 second interval between nares) with the mouse anesthetized in the supine position. Post-administration, mice remained anesthetized in the supine position for a minimum of 5 minutes to allow for uptake and prevent drainage. After 24 hours, 48 hours, 72 hours, 7 days, and 11 days post-immunization, the following tissues were excised and AF647 fluorescence (radiant efficiency) was measured by IVIS: nasal cavity (snout minus lower mandible), cervical lymph nodes, intestines, mesenteric lymph nodes, liver, and spleen. The nasal cavity was imaged by removing the head from the mouse body, then removing and discarding the lower mandible from the snout; images were collected of the underside ventral surface of the upper palate. To evaluate FcRn-dependence of amphiphile trafficking in the nasal mucosa, $FcRn^{-/-}$ mice were immunized intranasally with 5 µg AF647-amph-eOD combined with 5 µg SMNP and compared to WT mice (C57BL/6J) immunized intranasally with 5 µg AF647-amph-eOD or AF647-eOD combined with 5 µg SMNP. After 6, 24, or 72 hours post-immunization, the nasal cavity was isolated as described above and AF647 fluorescence (radiant efficiency) was measured by IVIS.

Histology and fluorescence microscopy of mouse nasal epithelium

Nasal cavity samples from FcRn$^{-/-}$ and C57BL/6 mice were processed for histology by FFPE (formalin fixed paraffin embedding) as follows: Samples were fixed in 10% neutral buffered formalin (NBF) for 24 hours at 25°C, then transferred into 70% ethanol for storage at 4°C. Fixed samples were decalcified in 10% EDTA disodium salt dihydrate (Sigma-Aldrich) at pH 7.4 for 10 days at 4°C, changing the EDTA solution every 3 days. Decalcified tissues were embedded in paraffin and sliced into 5µm coronal cross-sections using a microtome, starting 1mm in from the nares and proceeding at 500µm step intervals throughout the nasal cavity to a depth of 7.5mm. Sections located 1.5 to 3mm in from the nares were identified as the main site of vaccine deposition for detailed imaging. Slices were mounted on a glass slide and stained with DAPI using Vectashield HardSet Antifade Mounting Medium with DAPI (Vector Laboratories), then imaged using a Leica SP8 laser scanning confocal microscope with 25X water objective or 63X oil objective. Images were processed in ImageJ.

ELISA for albumin quantification

To assay albumin concentrations in the nasal mucosa, nasal wash was collected from C57BL/6 or FcRn$^{-/-}$ mice as described above. Concentration of albumin in the nasal secretions was measured using a commercial mouse albumin ELISA kit (Abcam, cat # ab207620) per the manufacturer's instructions.

Flow cytometry analysis of NALT uptake

BALB/c mice were immunized intranasally with 10µg AF647-eOD or AF647-amph-eOD combined with 5µg SMNP. One and four days later, mice were euthanized and the nasal-associated lymphoid tissue (NALT) was isolated by excising the upper palate *(90)* and processing to a single cell suspension as follows: The upper palate was enzymatically and mechanically digested in 1 ml RPMI-1640 containing 0.8 mg/ml collagenase/dispase (Roche) and 0.1 mg/ml DNase (Roche) by first cutting into less than 1mm chunks using fine-tipped spring-loaded scissors and then mashing in a 1.5ml biomasher tube (Kimble). After incubating for 15 minutes at 37°C with shaking, supernatant was removed and added to 10ml FACS buffer (PBS+1%BSA) at 4°C; the remaining tissue was subjected to a second round of digestion in 1 ml fresh enzyme mix for an additional 15 minutes at 37°C, then supernatant was removed and again added to cold FACS buffer. This FACS buffer solution was centrifuged at 500*xg* for 5 minutes to pellet cells; cells were washed once in FACS buffer, passed through a 70 µm filter, and finally centrifuged and resuspended in FACS buffer in a V-bottom plate for antibody staining.

Cells were washed with PBS and first stained with Live/Dead Near-IR (Invitrogen) at 1:500 in 100 µl PBS for 15 minutes at 25°C, then treated with anti-mouse CD16/32 Fc block (TruStain FcX, BioLegend) at 1:100 in 50 µl FACS buffer for 10 minutes at 4°C. To identify different cell populations with vaccine uptake, cells were stained with the following antibodies at a dilution of 1:100 in 50µl FACS buffer for 30 minutes at 4°C: anti-mouse CD3ε allophycocyanin (APC)-Cy7 (clone 145-2C11; BioLegend), B220 peridinin chlorophyll protein (PerCP)-Cy5.5 (RA3-6B2; BioLegend), CD45 brilliant ultraviolet (BUV) 737 (30-F11; BD Biosciences), major histocompatibility complex (MHC) II brilliant violet (BV) 605 (M5/114.15.2; BioLegend), CD11b BV421 (M1/70; BioLegend), CD11c BV510 (N418; BioLegend), F4/80 BV711 (BM8; BioLegend), CD103 phycoerythrin (PE) (2E7; BioLegend), CD8α BV786 (53-6.7; BD Biosciences), and CD169 PE-Cy7 (3D6.112; BioLegend). Cells were fixed with 2% paraformaldehyde and stored at 4°C until flow cytometry analysis. Counting beads (Invitrogen) were added prior to running on a BD LSR Fortessa.

Flow cytometry analysis of NALT GC B cell and Tfh cell responses

$FcRn^{-/-}$ and C57BL/6 mice were immunized intranasally with 5 µg eOD or amph-eOD combined with 5 µg SMNP. After 12 days, mice were euthanized and the NALT was isolated and processed as described above. Cells were washed with PBS and first stained with Live/Dead Aqua (Invitrogen) at 1:500 in 100 µl PBS for 15 minutes at 25°C, then treated with anti-mouse CD16/32 Fc block (TruStain FcX, BioLegend) at 1:100 in 50 µl FACS buffer for 10 minutes at 4°C. To identify eOD-specific germinal center (GC) B cells, half the cells from each NALT sample were stained with the following panel in 50µl FACS buffer for 30 minutes at 4°C: anti-mouse CD3ε BV711 at 1:200 (clone 145-2C11; BioLegend), B220 PE-Cy7 at 1:200 (RA3-6B2; BioLegend), CD38 FITC at 1:200 (90; BioLegend), GL7 PerCP-Cy5.5 at 1:150 (GL7; BioLegend), eOD-tetramer PE at 1:100, and eOD-tetramer BV421 at 1:50. Fluorophore-labeled eOD tetramers were prepared by first reacting eOD with maleimide-PEG2-biotin (Thermo Fisher Scientific) per the manufacturer's instructions, and then complexing 5 molar equivalents of biotinylated-eOD with 1 equivalent of streptavidin-PE or streptavidin-BV421 (BioLegend) for 30 minutes at 25°C. To identify T follicular helper (Tfh) cells, half the cells from each NALT sample were stained with the following antibodies in 50 µl FACS buffer for 30 minutes at 4°C: anti-mouse B220 BV510 at 1:200 (clone RA3-6B2; BioLegend), CD4 BV711 at 1:200 (GK1.5; BioLegend), CD44 PE-Cy7 at 1:200 (IM7; BioLegend), Inducible T Cell Costimulator (ICOS) PE at 1:100 (7E.17G9; BioLegend), programmed cell death protein 1 (PD-1) BV650 at 1:50 (J43; BD Biosciences), and CXCR5-biotin at 1:50 (2G8; BD Biosciences) followed by streptavidin-BV421 at 1:100 (BioLegend).

Mouse sample collection

Vaginal mucosal fluid was collected from anesthetized mice by vaginal lavage using 75 µl sterile PBS (3x25 µl instillations, each aspirated three to five times) combined with 5 µl of 25X protease inhibitor (EDTA-free SIGMAFAST Protease Inhibitor Cocktail Tablets, Sigma-Aldrich); fluid was centrifuged at 12,000x*g* for 10 minutes at 4°C to collect supernatant. Fecal wash was collected from mouse fecal pellets (4 pellets of ~0.75cm each per mouse) combined with 300 µl 1X protease inhibitor; samples were vortexed, incubated for 1 hour at 4°C, vortexed a second time, then centrifuged at 13,000x*g* for 15 minutes at 4°C to collect supernatant. Saliva wash was collected by dispensing 30 µl sterile PBS between the mouse's cheek and gumline (aspirated three to five times), repeated on both sides, and combined with 10 µl of 2X protease inhibitor. All fluid samples were stored in aliquots at -80°C for future analysis.

Post-euthanasia, BM and FRT tissues were collected to evaluate immune memory and resident plasma cell responses in the vaginal mucosa. FRT was isolated from the vaginal opening to the ovaries, cut into 1 to 3 mm chunks using fine-tipped spring-loaded scissors, and digested in 2 ml/sample of RPMI-1640 containing 2 mg/ml collagenase D (Roche), 0.6 U/ml Dispase II (StemCell Technologies), and 0.2 mg/ml DNase I (Roche) for 30 minutes at 37°C with shaking. Samples were then centrifuged at 500xg for 5 minutes to pellet tissue and cells, supernatant discarded, and resuspended in 2ml fresh digestion media for an additional incubation for 30 minutes at 37°C with shaking. The digestion was quenched by adding an equal volume of RPMI-1640 containing 10% fetal bovine serum (FBS) and 1% penicillin/streptomycin. This solution plus remaining tissue was passed through a 70 µm cell strainer using the plunger end of a 1-ml syringe for additional mechanical digestion, then centrifuged at 500x*g* for 5 minutes and resuspended in 5 ml ACK lysis buffer for 5 minutes at 4°C to lyse residual red blood cells. An equal volume of cRPMI was added to quench the ACK buffer; samples were then centrifuged at 500x*g* for 5 minutes and rinsed once with cRPMI, passed through a 70 µm filter a second time, and finally

centrifuged and resuspended a final time in cRPMI for counting and further analysis by enzyme-linked immunosorbent spot (ELISPOT) assay or flow cytometry.

For RBD studies, nasal wash and bronchoalveolar lavage fluid (BALF) were collected to evaluate resident mucosal antibody responses in the upper and lower respiratory tract. Nasal wash was collected from 2x15 µl instillations of PBS, one in each nare (aspirated three to five times), combined with 10µl of 2X protease inhibitor. BALF was collected from 2x1 ml instillations of sterile PBS in the lungs using a 24G x ¾" catheter through the trachea. Both fluid samples were centrifuged at 12,000x*g* for 10 min at 4°C to collect supernatant, then stored at -80°C.

ELISA analyses of murine antibody titers

Anti-eOD and anti-RBD IgG and IgA binding titers were measured in mouse serum and mucosal samples (vaginal wash, fecal wash, saliva, nasal wash, and BALF) by ELISA. To capture eOD-specific antibodies from immunized mice, MAXIsorp (Thermo Fisher Scientific) 96-well plates were coated directly with eOD antigen at 2 µg/ml in PBS overnight at 4°C. To capture RBD-specific antibodies, Costar Polystyrene High Binding 96-well plates (Corning) were coated directly with RBD antigen at 2 µg/ml in PBS overnight at 4°C. Plates were then blocked with PBS + 2% BSA for 2 hours at 25°C. Mouse serum samples were diluted in block buffer (PBS + 2% BSA) starting at 1:100 or 1:200, and mucosal samples were diluted in block buffer starting at 1:10, followed by 4X serial dilutions. For eOD ELISAs, VRC01 at 5 µg/ml was used as a positive control; for RBD ELISAs, mAb CR3022 or Fc-fusion protein ACE2-Fc at 5µg/ml were used as positive controls. Samples were incubated in plates for 2 hours at 25°C, followed by detection with 1:5000 goat anti-mouse IgG-horseradish peroxidase (HRP, BioRad) or 1:2000 goat anti-mouse IgA-HRP (Invitrogen) in block buffer for 1 hour. Plates were developed using 3,3',5,5'-Tetramethylbenzidine (TMB) substrate for 1 to 20 minutes and stopped with 2N sulfuric acid, and the resulting absorbance (A450/A540) was measured on a plate reader. For all titer analyses, samples directly compared across groups were developed for the same amount of time. Cut-off titers are reported as inverse dilutions giving an HRP absorbance (A450 – A540) of 0.2 (RBD) or 0.1 (eOD) based on background.

ELISPOT analysis of mouse plasma cells

IgG and IgA plasma cells were analyzed in BM and FRT tissue at 35 or greater than 52 weeks post-prime, as indicated, using PVDF-MSIP filter plates (0.45 µm High Protein Binding Immobilon-P Membrane filter plates, Millipore) and Mouse IgG/A ELISpot-BASIC kits (Mabtech). To quantify eOD antigen-specific IgG and IgA plasma cells, filter plates were coated with 10 µg/ml eOD in 100 µl sterile PBS and incubated overnight at 4°C; cells were plated at 500,000 and 250,000 cells per well in 100 µl cRPMI. To quantify total IgG and IgA plasma cells, filter plates were coated with 15 µg/ml anti-IgG (purified goat anti-mouse IgG capture antibody, Mabtech) or anti-IgA (monoclonal antibody MT45A, Mabtech), respectively, in 100 µl sterile PBS and incubated overnight at 4°C; cells were plated at 100,000 and 50,000 cells per well in 100 µl cRPMI. Plates were then incubated for 18 to 20 hours at 37°C; spot detection was carried out per manufacturer instructions, and plates were read on a CTL ImmunoSpot Analyzer.

Mouse parenteral control immunization

To compare intranasal immunization to a parenteral control, BALB/c mice were immunized intranasally or subcutaneously at the scruff of the neck with 5 µg amph-eOD combined with 25

µg cdGMP. Mice were primed on day 0 and boosted on day 42. Blood, vaginal, and fecal samples were collected at regular intervals as described above.

ELISA for anti-PEG antibodies

Antibody responses to PEG included in the amph-protein conjugates was assayed by ELISA. Briefly, MaxiSorp ELISA plates were coated with streptavidin at 1 µg/mL in PBS for 4 hours at 25°C, blocked with PBS + 2% bovine serum albumin (BSA) overnight at 4°C, then washed three times with wash buffer (PBS containing 0.2% Tween20). Biotin-PEG-OH (Creative PEGWorks, cat. #PJK-1946) was added to the plates in blocking buffer (1 µg/mL) and incubated for 2 hours at 25°C. After washing plates three times with wash buffer, mouse serum samples and mouse anti-PEG IgG standard antibody (AffinityImmuno kit cat. #EL-141-PEG-mIGG, starting at 1 µg/ml followed by 2X serial dilutions) were added and incubated for 2 hours prior to washing. Anti-mouse IgG-HRP diluted 1:5000 in blocking buffer was used as a detection antibody. Samples were incubated for 1 hour at 25°C before washing and adding TMB substrate, followed by 2 N H_2SO_4 as a stop solution. Absorbance was measured at 450 nm.

ACE2:RBD binding inhibition assay

Functional antibody inhibition of ACE2:RBD binding was measured in mouse serum and BALF as a preliminary indication of neutralizing antibodies using SARS-CoV-2 Surrogate Virus Neutralization Test Kits (Genscript), per manufacturer instructions. Mouse serum was diluted starting at 1:10 and BALF was diluted 1:2, followed by 4X serial dilutions. Inhibition (IC_{50}) was defined as the sample dilution at which 50% reduction in ACE2:RBD binding was observed relative to a negative control (no inhibition).

Pseudovirus-based SARS-CoV-2 neutralization assay

The SARS-CoV-2 pseudoviruses expressing a luciferase reporter gene were generated in an approach similar to those described previously *(91, 92)*. Briefly, the packaging plasmid psPAX2 (AIDS Resource and Reagent Program), luciferase reporter plasmid pLenti-CMV Puro-Luc (Addgene), and spike protein-expressing pcDNA3.1-SARS CoV-2 SΔCT were co-transfected into HEK293T cells by lipofectamine 2000 (Thermo Fisher Scientific). The supernatants containing the pseudotype viruses were collected 48 hours post-transfection, which were purified by centrifugation and filtration with 0.45 µm filter. To determine the neutralization activity of mouse serum and mucosal samples, HEK293T expressing human ACE2 (hACE2) were seeded in 96-well tissue culture plates at a density of 1.75 x 10^4 cells per well overnight. Samples (serum, saliva, nasal wash, vaginal wash, fecal wash, and BALF) were first heat-inactivated at 56°C for 30 minutes. Three-fold serial dilutions of heat-inactivated serum or mucosal samples were then prepared and mixed with 50 µL of pseudovirus. The mixture was incubated at 37°C for 1 hour before adding to HEK293T-hACE2 cells. At 48 hours after infection, cells were lysed in Steady-Glo Luciferase Assay (Promega) according to the manufacturer's instructions. SARS-CoV-2 neutralization titers (NT_{50}) were defined as the sample dilution at which a 50% reduction in relative light unit (RLU) was observed relative to the average of the virus control wells.

Non-human primates

Six female Indian rhesus macaques (Macaca mulatta) were assigned to the IVIS trafficking study (n = 3 animals per group). Twelve female Indian rhesus macaques between 3 and 4 years of age

were assigned to the longitudinal immunization study (n = 6 animals per group). Macaques were distributed such that age, weight, and MHC genotyping were equivalent across groups. Animals were housed and maintained at the New Iberia Research Center (NIRC) of the University of Louisiana at Lafayette in accordance with the rules and regulations of the “Guide for the Care and Use of Laboratory Animals”. The entire study (protocol 8789-08) was reviewed and approved by the University of Louisiana at Lafayette IACUC. All animals were negative for SIV, simian T cell leukemia virus and simian retrovirus. The animals were also typed for MHC and those expressing the MamuB*008 or B*017 alleles were excluded whereas those expressing the MamuA*001 allele were distributed equally among the groups.

IVIS trafficking in non-human primate tissues

In vivo trafficking of AF647-labeled amph-eOD and eOD was evaluated following intranasal administration using an IVIS fluorescence imaging system (Perkin Elmer). Macaques were immunized intranasally in a dropwise manner directly to each nostril, 200 µl per nare (400 µl total per animal), with 100 µg AF647-amph-eOD or AF647-eOD mixed with 375 µg SMNP. Post-administration, animals remained in the supine position under anesthesia for 10 minutes to allow for vaccine uptake and to prevent drainage. After 24 hours, the tonsils, adenoids, cervical lymph nodes, axillary lymph nodes, and nasal tissue including turbinates were collected, fixed in 4% paraformaldehyde for 5 days, then transferred to PBS + 0.1% paraformaldehyde + 0.05% sodium azide for storage at 4°C prior to evaluation by IVIS.

ELISA analysis of non-human primate antibody titers

To measure eOD-specific antibody titers, MAXIsorp 96-well plates (Thermo Fisher Scientific) were coated with 2 µg/mL of gp120 eOD monomer in PBS. Serum samples were diluted 1:50 and mucosal washes were diluted 1:10 in 2% BSA block buffer, followed by 4X serial dilutions. hVRC01 at 5µg/ml was included as a positive control. Samples were incubated for 2 hours at room temperature, followed by detection with 1:5000 goat anti-human IgG-HRP (Jackson ImmunoResearch) or 1:2000 goat-anti-human IgA-HRP (Thermo Fisher Scientific). Cut-off titers are reported as inverse dilutions giving an HRP absorbance (A450 – A540) of 0.2 (IgA) or 0.1 (IgG) based on background.

ELISPOT analysis of non-human primate plasma cells

Total and antigen-specific plasmablast responses in peripheral blood were determined by ELISPOT assay as previously described *(94)*. Briefly, 96-well multiscreen HTS filter plates (Millipore) were coated overnight at 4°C with 100 µl/well of 5 µg/ml of goat anti-monkey IgG, IgM or IgA antibodies (Rockland) or of 1 µg/ml of HIV eOD-gp120, respectively. Plates were washed with PBS plus 0.05% Tween 20 (PBS-T) and blocked with complete medium at 37°C for 2 hours. Freshly isolated cells were plated in duplicates in three-fold serial dilutions and incubated overnight in a 5% CO_2 incubator at 37°C. Plates were washed with PBS-T and incubated with biotin-conjugated anti-monkey IgG, IgM or IgA antibodies (Rockland) diluted 1:1000 for 1 hour at 37°C. After washing, plates were incubated with HRP-conjugated streptavidin diluted 1:1,000 (Vector Labs) at 25°C for 2 hours and developed using the AEC substrate kit (BD Biosciences). To stop the reaction, plates were washed extensively with water followed by air drying. Spots were imaged and counted using an Immunospot ELISPOT Analyzer (Cellular Technology Limited); the number of spots specific for each Ig isotype was reported as the number of either total or antigen-specific antibody-producing cells per million PBMCs.

Figures

A

eOD-PADRE:
ETG**C**HHHHHHGGDTITLPCRPAPPPHCSSNITGLILTRQG
GYSNDNTVIFRPSGGDWRDIARCQIAGTVVSTQLFLNGSL
AEEEVVIRSEDWRDNAKSICVQLNTSVEINCTGAGHCNIS
RAKWNNTLKQIASKLREQYGNKTIIFKPSSGGDPEFVNHS
FNCGGEFFYCDSTQLFNSTWFNSTGSAKFVAAWTLKAAA

B

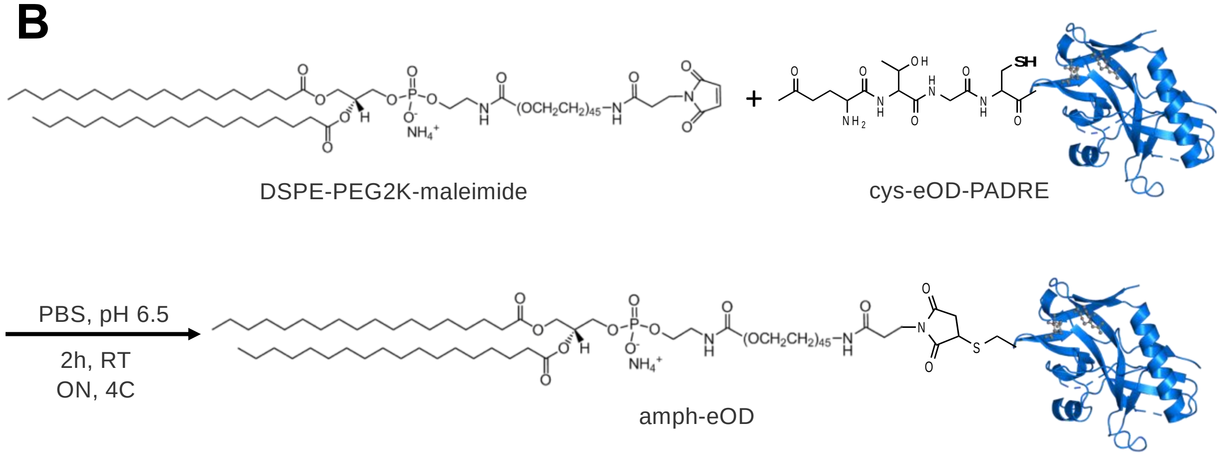

Figure S1. Synthesis of amphiphile-protein conjugates. (**A**) The sequence of eOD protein is shown with pan HLA DR-binding epitope (PADRE) peptide in red. (**B**) The reaction scheme for preparation of amph-eOD antigen conjugate is shown.

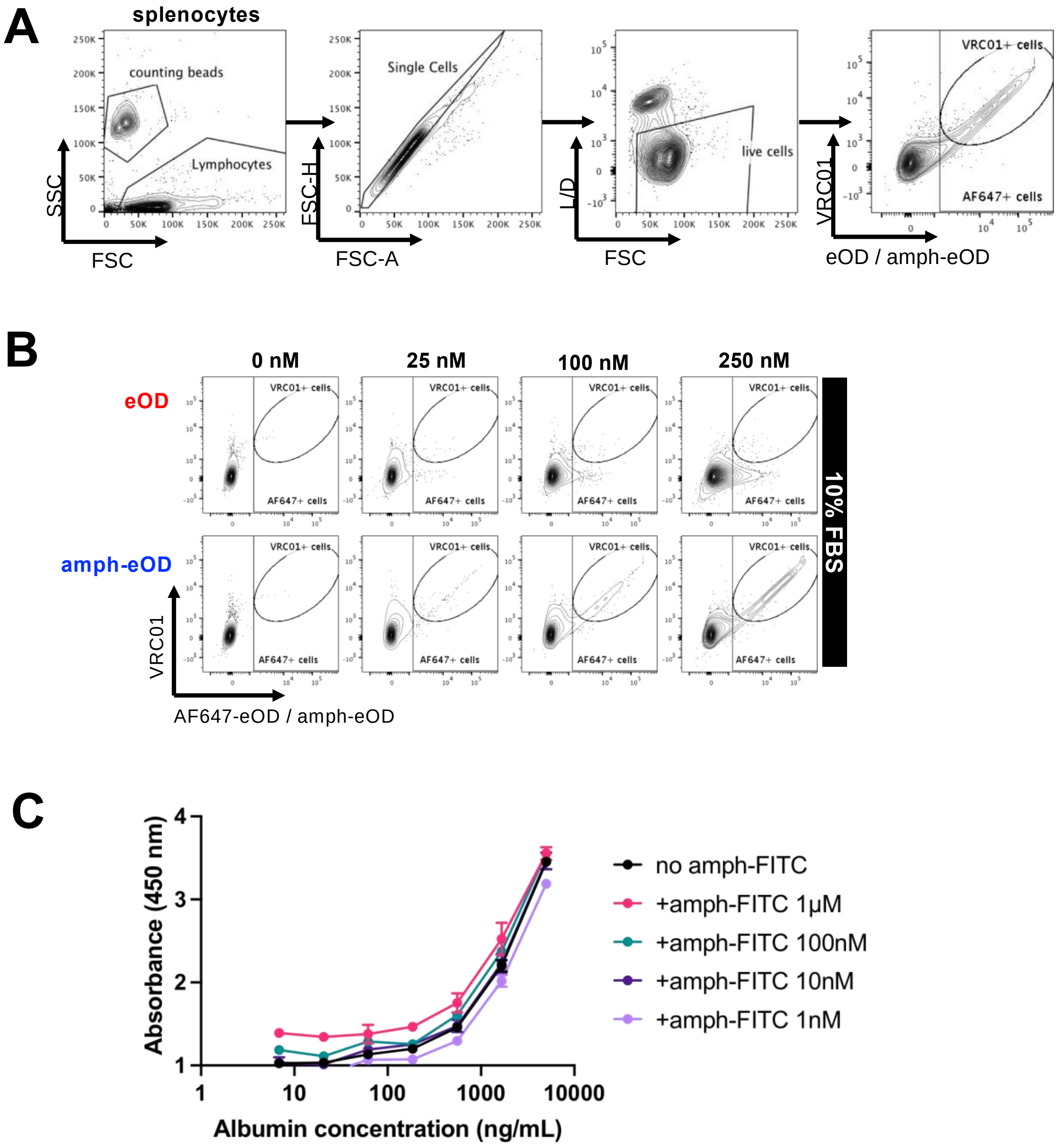

Figure S2. Amph-protein conjugate insertion into cell membranes. AF647-labeled eOD or amph-eOD were incubated with murine C57BL/6 splenocytes for 1 hour at 37°C at a range of concentrations, washed, and stained with Rhodamine-labeled VRC01 antibody. Samples were evaluated by flow cytometry to assess eOD versus amph-eOD cell membrane insertion. (**A**) The gating strategy is shown for identification of VRC01^{+} and AF647^{+} cells; FSC, forward scatter; SSC, side scatter; A, area; H, height; L/D, live/dead dye. (**B**) representative flow cytometry plots of eOD/amph-eOD and VRC01 binding to the cells at varying concentrations of eOD are shown. FBS, fetal bovine serum. (**C**) ELISA measurements are shown for human serum albumin binding to plate-bound human FcRn in the presence of varying concentrations of DSPE-PEG$_{2K}$-FITC.

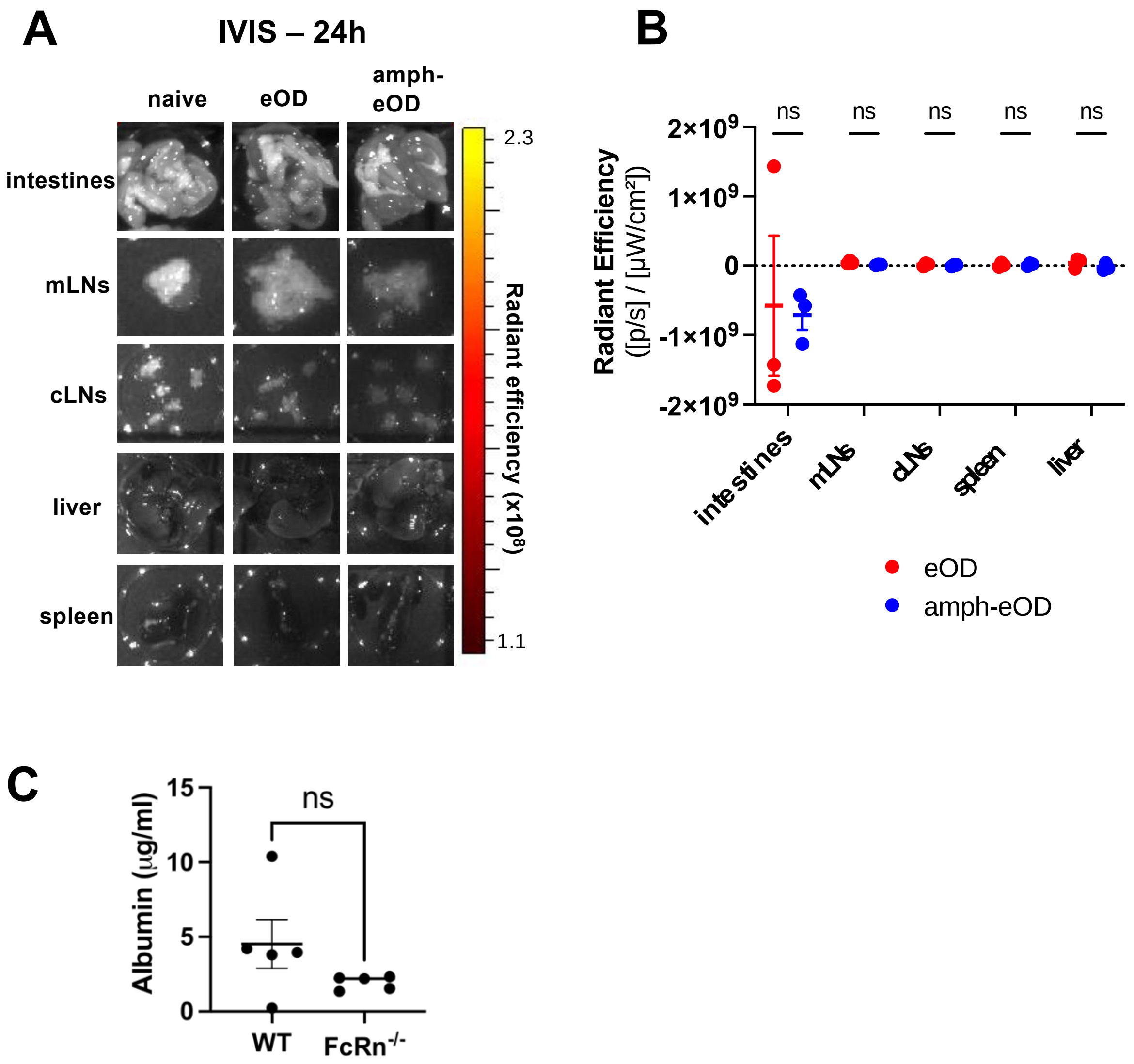

Figure S3. Systemic distribution of amph-protein conjugates in mice. BALB/c mice (n = 3 animals per group) were immunized intranasally with 5 µg AF647-eOD or AF647-amph-eOD mixed with 5 µg SMNP adjuvant, and tissues were collected after 24 hours for IVIS analysis of AF647 fluorescent signal to evaluate systemic dissemination and distal lymphatic drainage of eOD versus amph-eOD. (**A**) Representative IVIS images and (**B**) quantified IVIS signal are shown in the intestines, mesenteric lymph nodes (mLNs), cervical lymph nodes (cLNs), liver, and spleen after 24 hours. Statistical comparison was performed using a Mann-Whitney U test followed by Holm-Sidak correction with α=0.05. (**C**) ELISA analysis is shown for albumin concentrations in the nasal wash of wild-type (WT) versus $FcRn^{-/-}$ mice (n = 5 animals per group). Statistical comparison was performed using Welch's t-test. All data showing mean ± standard error of the mean (s.e.m.). ns, not significant.

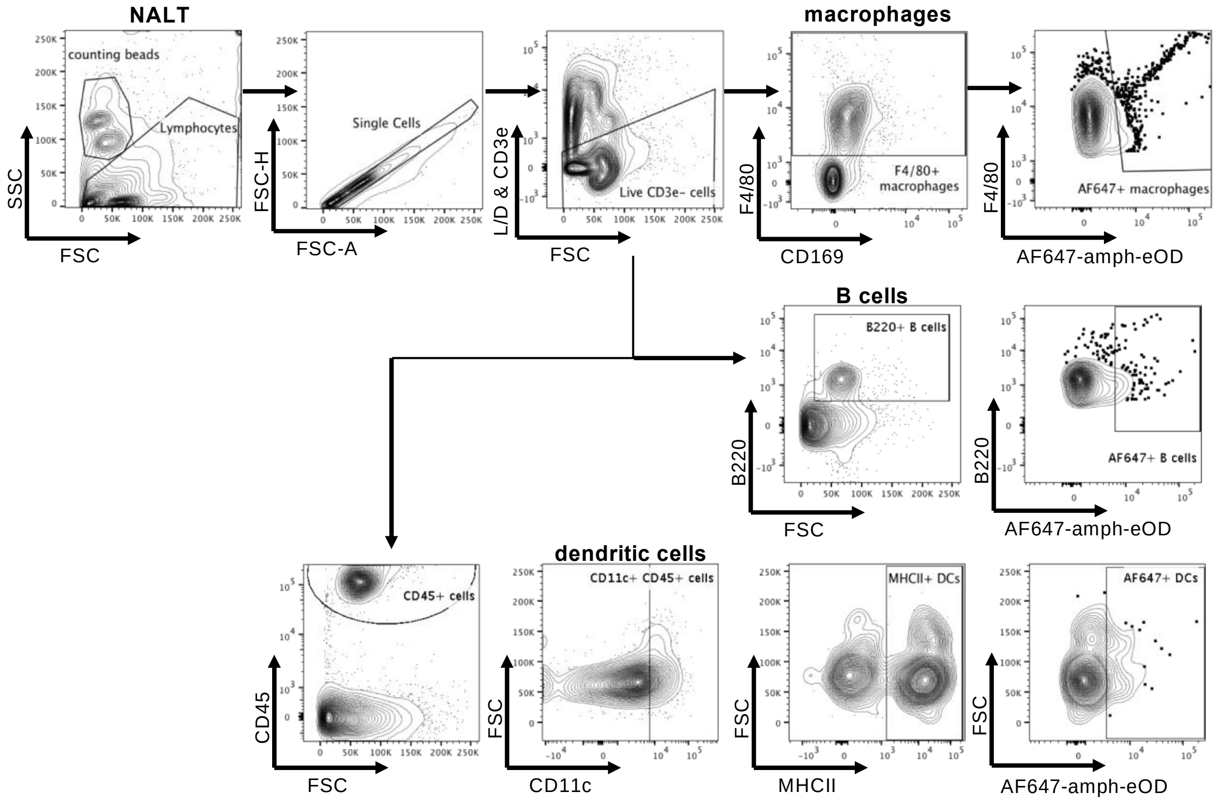

Figure S4. Amph-protein uptake in mouse NALT cell populations. Groups of BALB/c mice (*n* = 5 animals per group) were immunized intranasally with 10 µg AF647-amph-eOD or AF647-eOD mixed with 5 µg SMNP adjuvant, and NALT tissue was isolated 1 or 4 days later for flow cytometry analysis of antigen uptake. Schematic shows gating strategy to identify AF647-labeled vaccine uptake in macrophages, B cells, and dendritic cells of the NALT. MHC, major histocompatibility complex.

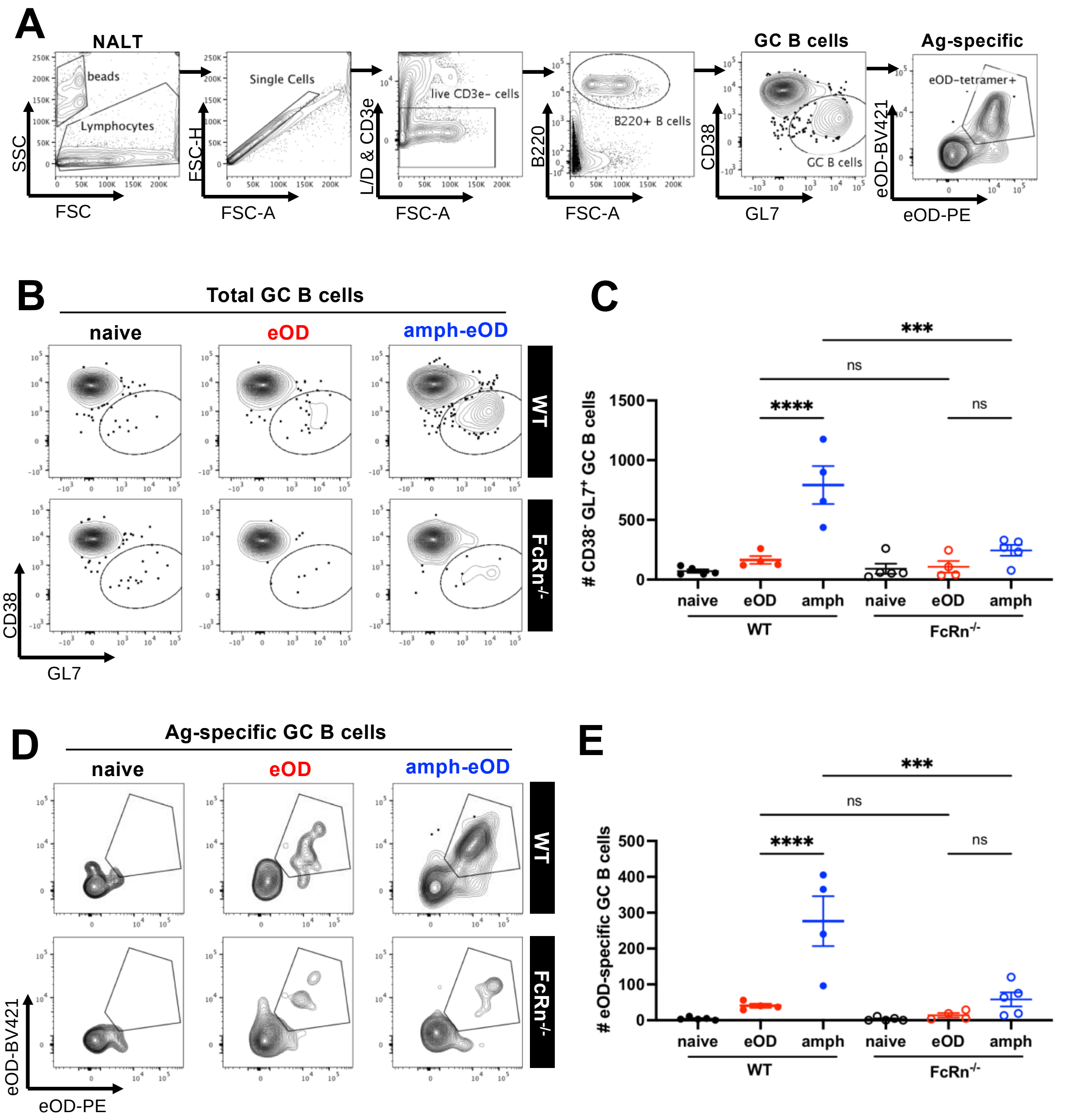

Figure S5. GC B cell responses in mouse NALT following intranasal immunization with amph-protein. Groups of C57BL/6 (WT) or FcRn$^{-/-}$ mice (n = 5 animals per group) were immunized with 5 µg eOD or amph-eOD mixed with 5 µg SMNP adjuvant, and GC responses were analyzed by flow cytometry on day 12. (**A**) The gating strategy for identification of GC B cells is shown. (**B**) Representative flow cytometry plots and (**C**) absolute number of cells are presented, showing total CD38^{-}GL7^{+} GC B cells for all NALT samples, including controls. (**D**) Representative flow cytometry plots and (**E**) absolute number of cells are presented, showing eOD-tetramer^{+} GC B cells for all NALT samples, including controls. Statistical significance was determined using one-way ANOVA followed by Tukey's post-hoc test. ***p<0.001, ****p<0.0001; ns, not significant. All data show mean ± s.e.m.

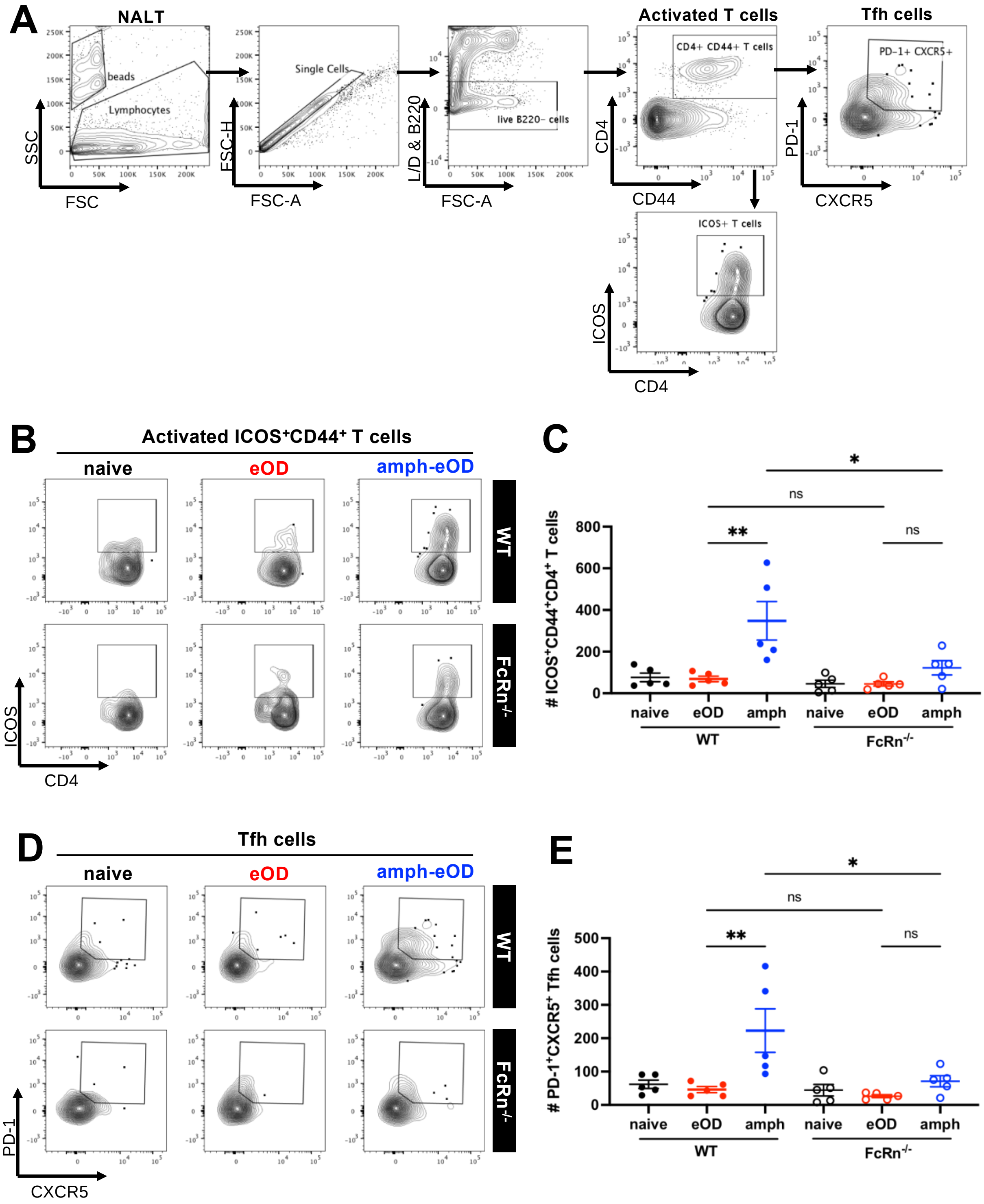

Figure S6. Tfh cell responses in mouse NALT following intranasal immunization with amph-protein. Groups of C57BL/6 (WT) or FcRn$^{-/-}$ mice (n = 5 animals per group) were immunized with 5 µg eOD or amph-eOD mixed with 5 µg SMNP adjuvant, and T_{FH} responses were analyzed by flow cytometry on day 12. (**A**) The gating strategy for identification of Tfh cells

is shown. (**B**) Representative flow cytometry plots and (**C**) absolute number of cells are presented, showing activated $ICOS^+CD4^+CD44^+$ T cells for all NALT samples, including controls. (**D**) Representative flow cytometry plots and (**E**) absolute number of cells are presented, showing $PD\text{-}1^+CXCR5^+$ Tfh cells for all NALT samples, including controls. Statistical significance was determined using one-way ANOVA followed by Tukey's post-hoc test.*p<0.05, **p<0.01; ns, not significant. All data showing mean ± s.e.m.

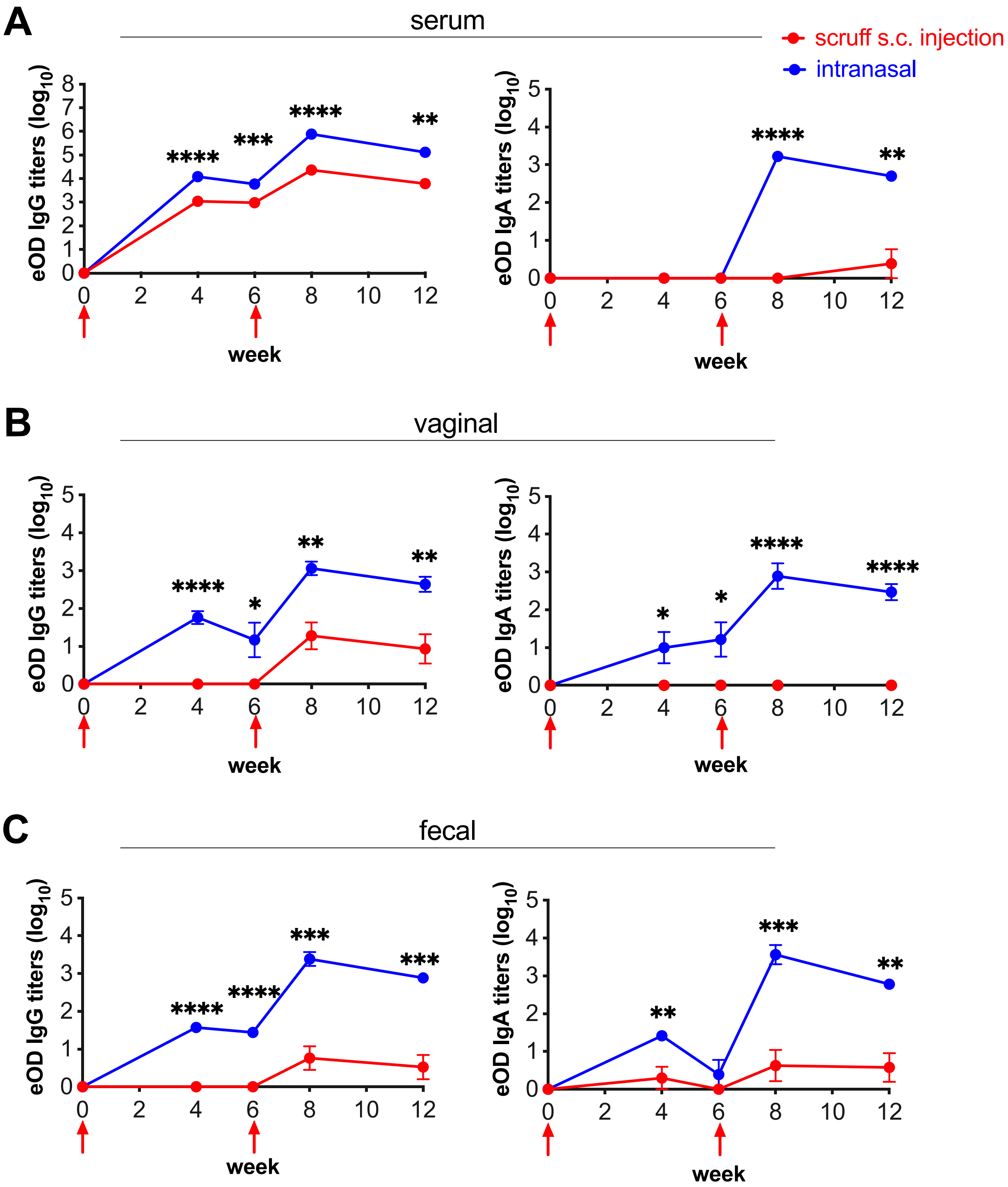

Figure S7. Control parenteral immunization with amph-protein conjugate elicits negligible mucosal antibody response compared to intranasal immunization. BALB/c mice (n = 5 animals per group) were immunized intranasally (i.n.) or subcutaneously (s.c.) injected at the scruff with 5 µg amph-eOD mixed with 25 µg cdGMP adjuvant and boosted 6 weeks later with the same formulation (red arrows). IgG and IgA titers were measured in the (**A**) serum, (**B**) vaginal wash, and (**C**) feces. Statistical significance comparing i.n. and s.c. groups was determined by

unpaired t-test. *$p<0.05$, **$p<0.01$, ***$p<0.001$, ****$p<0.0001$. All data are presented as mean ± s.e.m.

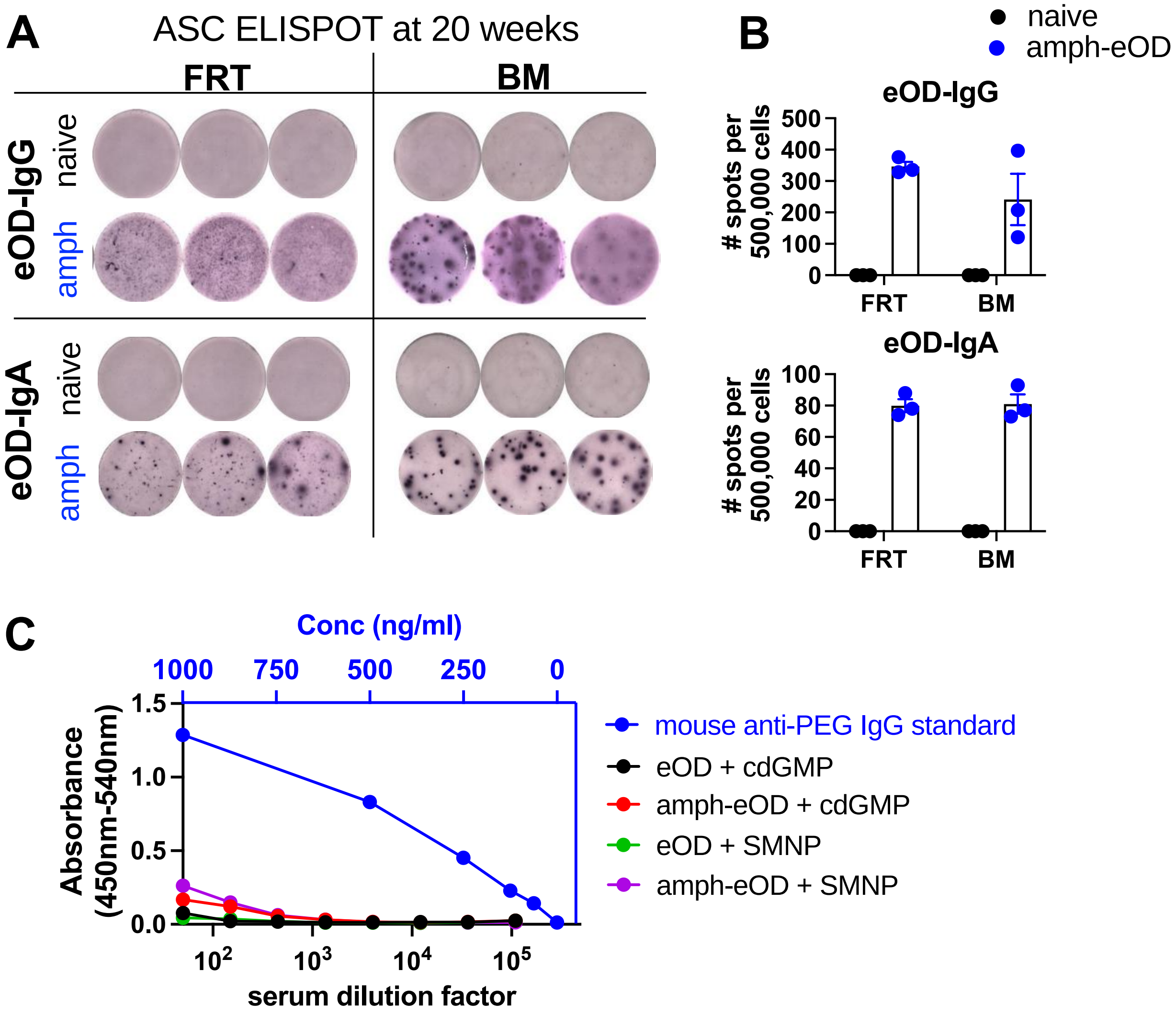

Figure S8. Long-lived antigen-specific IgG and IgA plasma cells are established in mice following intranasal immunization with amph-protein, without induction of anti-PEG antibodies. BALB/c mice (*n* = 3 animals per group) were immunized i.n. with 5 µg amph-eOD mixed with 25 µg cdGMP adjuvant and boosted 6 weeks later with the same formulation. Female reproductive tract (FRT) and bone marrow (BM) eOD-specific IgG and IgA antibody-secreting cells (ASCs) were assessed by ELISPOT at 20 weeks post immunization. (**A**) Representative well images and (**B**) quantified number of antibody-secreting plasma cells per 500,000 cells are shown. All data are presented as mean ± s.e.m. (**C**) Serum samples from mice immunized as in Fig. 4A and F with saponin (collected at week 11) or cdGMP adjuvants (collected at week 12) were analyzed by ELISA for anti-PEG IgG, comparing to a reference anti-PEG IgG standard.

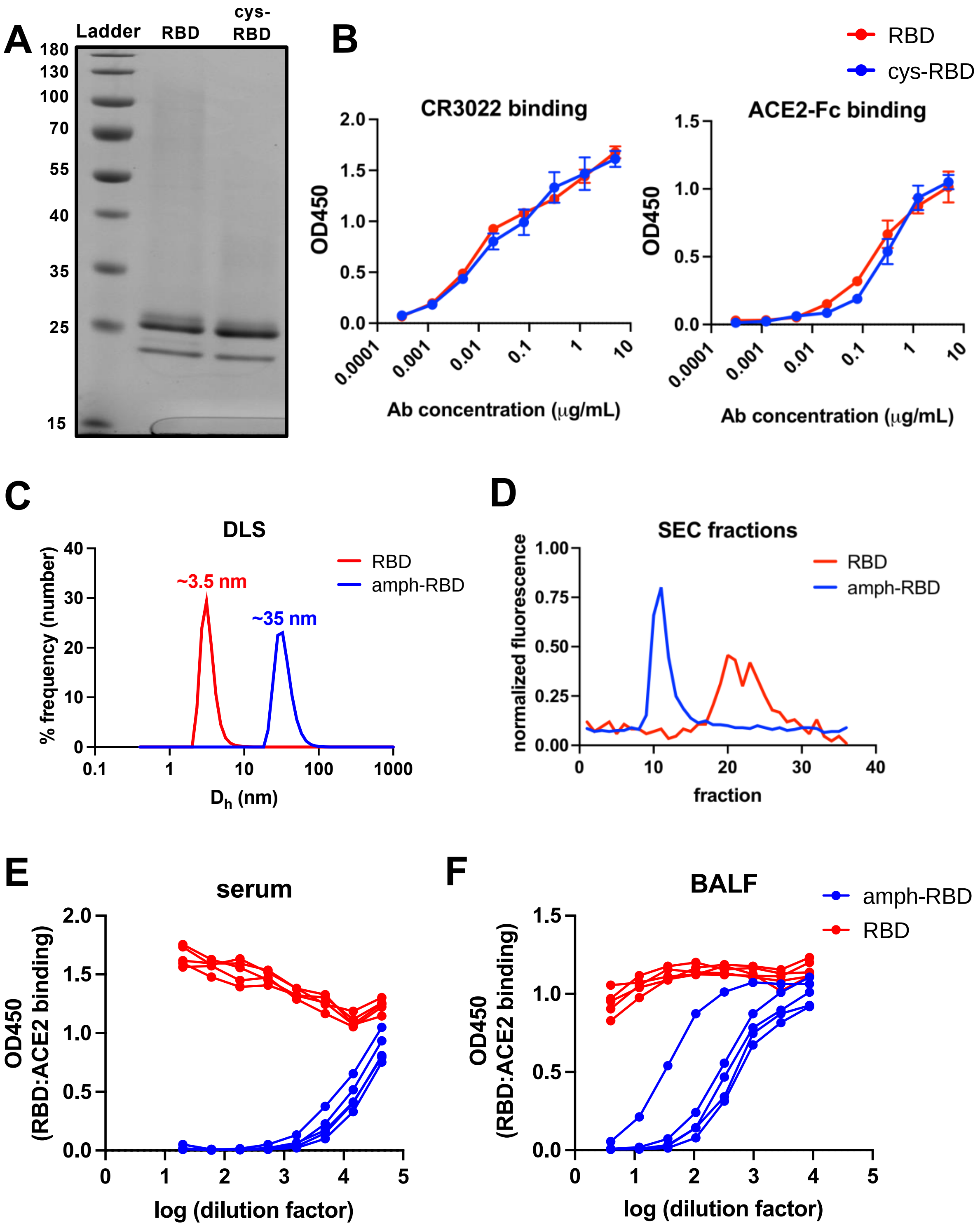

Figure S9. Synthesis and characterization of amph-RBD. (**A**) A gel comparing RBD versus cys-RBD is shown. (**B**) Antigenicity ELISA results comparing binding of RBD versus cys-RBD to monoclonal antibodies CR3022 and angiotensin converting enzyme 2 (ACE2)-Fc are shown. (**C**) Dynamic light scattering (DLS) analysis of RBD and amph-RBD is shown as number-weighted % frequency. D_h, hydrodynamic diameter. (**D**) The size exclusion chromatography (SEC) profile of

RBD versus amph-RBD is shown. (**E and F**) ACE2 binding inhibition raw absorbance curves for week 6 serum (E) and bronchoalveolar lavage fluid (BALF) (F) were used to determine half-maximal inhibitory concentration (IC_{50}) values shown in Fig. 5E. All data are presented as mean ± s.e.m.

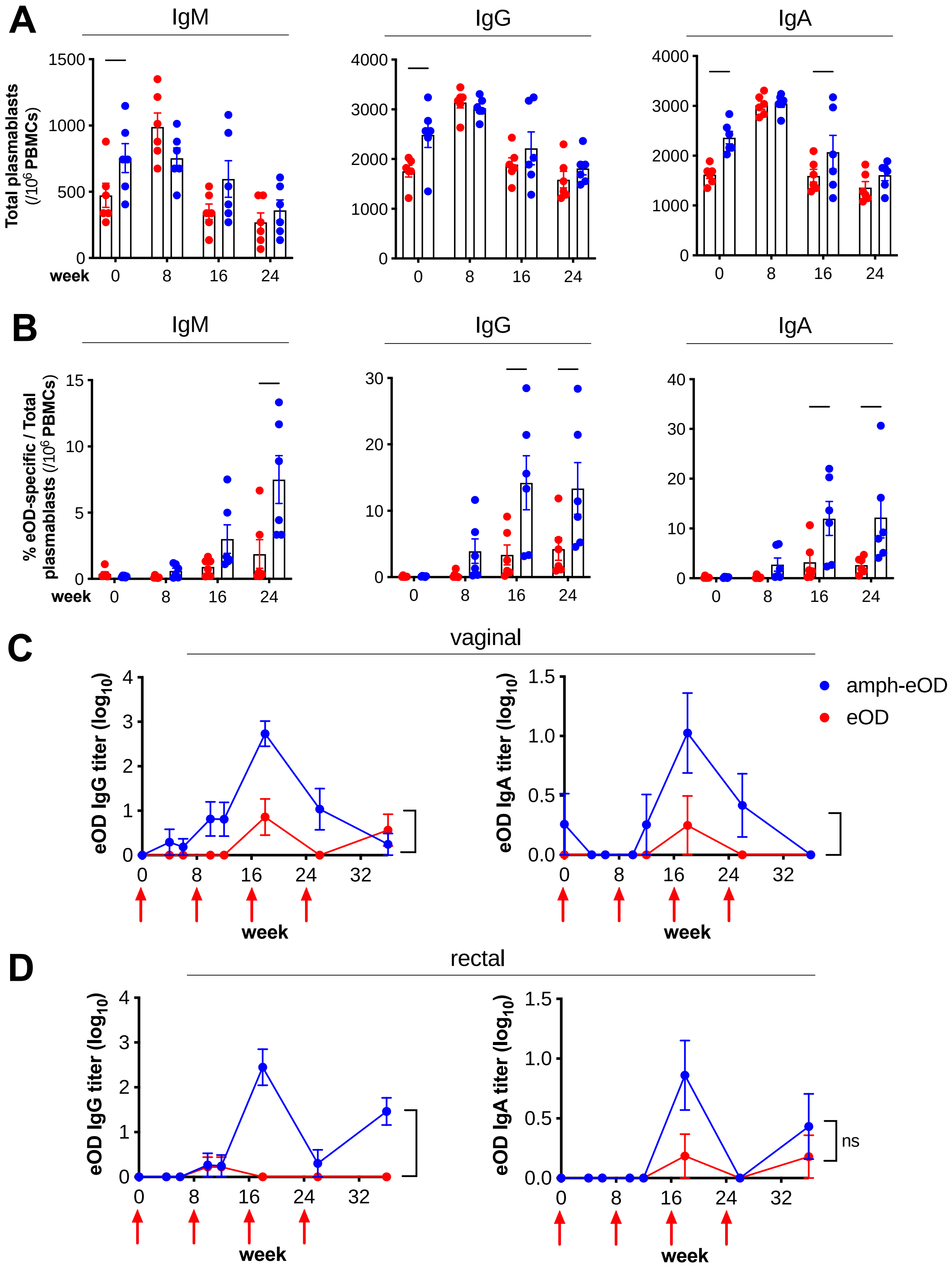
A
IgM
IgG
IgA
Total plasmablasts (/10^6 PBMCs)
week
0
8
16
24
B
% eOD-specific / Total plasmablasts (/10^6 PBMCs)
C
vaginal
eOD IgG titer (log10)
eOD IgA titer (log10)
amph-eOD
eOD
week
D
rectal
ns

Figure S10. Intranasal immunization with amph-protein conjugates leads to improved humoral immune responses in non-human primates. Rhesus macaques (*n* = 6 female animals per group) were immunized i.n. with 100 µg eOD or amph-eOD mixed with 375 µg SMNP adjuvant and boosted at 8, 16, and 24 weeks with the same formulations (red arrows in C and D). (**A**) Total IgM, IgG, and IgA secreting plasmablasts frequencies and (**B**) Percent antigen-specific IgM, IgG, and IgA secreting plasmablasts in peripheral blood were determined by ELISPOT; data are shown as a percent of total peripheral blood mononuclear cells (PBMCs). Statistical significance for (A) and (B) was determined using multiple unpaired t-tests. (**C**) Vaginal IgG and IgA titers were measured over time; (**D**) rectal IgG and IgA titers were measured over time. Statistical significance for (C) and (D) was determined using a two-way ANOVA comparing eOD and amph-eOD across all timepoints. *p<0.05, **p<0.01, ***p<0.001, ****p<0.0001; ns, not significant. All data are presented as mean ± s.e.m.

Supplementary Data Files

Data File S1. Tabulated individual-level data for main manuscript figures.

Data File S2. Tabulated individual-level data for supplementary figures.
